# Pyrazoleamide compounds are potent antimalarials that target Na^+^ homeostasis in intraerythrocytic *Plasmodium falciparum*

**DOI:** 10.1038/ncomms6521

**Published:** 2014-11-25

**Authors:** Akhil B. Vaidya, Joanne M. Morrisey, Zhongsheng Zhang, Sudipta Das, Thomas M. Daly, Thomas D. Otto, Natalie J. Spillman, Matthew Wyvratt, Peter Siegl, Jutta Marfurt, Grennady Wirjanata, Boni F. Sebayang, Ric N. Price, Arnab Chatterjee, Advait Nagle, Marcin Stasiak, Susan A. Charman, Iñigo Angulo-Barturen, Santiago Ferrer, María Belén Jiménez-Díaz, María Santos Martínez, Francisco Javier Gamo, Vicky M. Avery, Andrea Ruecker, Michael Delves, Kiaran Kirk, Matthew Berriman, Sandhya Kortagere, Jeremy Burrows, Erkang Fan, Lawrence W. Bergman

**Affiliations:** 1 Department of Microbiology and Immunology, Center for Molecular Parasitology, Drexel University College of Medicine, 2900 Queen Lane, Philadelphia, Pennsylvania 190129, USA; 2 Department of Biochemistry, University of Washington, Box 357350, Seattle, Washington 98195, USA; 3 Wellcome Trust Sanger Institute, Hinxton, Cambridge CB101SA, UK; 4 Research School of Biology, The Australian National University, Canberra, Australian Capital Territory 0200, Australia; 5 Medicines for Malaria Venture, PO Box 1826, 20Rt de Pr-Bois, Geneva 15 1215, Switzerland; 6 Division of Global and Tropical Health, Menzies School of Health Research and Charles Darwin University, PO Box 41096, Casuarina, Northern Territory 0811, Australia; 7 Eijkman Institute for Molecular Biology, Jl. Diponegoro 69, Jakarta 10430, Indonesia; 8 Nuffield Department of Clinical Medicine, Centre for Tropical Medicine, University of Oxford, Oxford OX3 7LJ, UK; 9 Genomics Institute of the Novartis Research Foundation, 10675 John Jay Hopkins Drive, San Diego, California 92121, USA; 10 Center for Drug Candidate Optimisation, Monash University, 381 Royal Parade, Parkville, Victoria 3052, Australia; 11 GlaxoSmithKline, Malaria Support Group, Calle Severo Ochoa 2, Tres Cantos 28760, Spain; 12 Eskitis Institute, Griffith University, Don Young Road, Nathan, Queensland 4111, Australia; 13 Department of Life Sciences, South Kensington Campus, Imperial College, London SW7 2AZ, UK

## Abstract

The quest for new antimalarial drugs, especially those with novel modes of action, is essential in the face of emerging drug-resistant parasites. Here we describe a new chemical class of molecules, pyrazoleamides, with potent activity against human malaria parasites and showing remarkably rapid parasite clearance in an *in vivo* model. Investigations involving pyrazoleamide-resistant parasites, whole-genome sequencing and gene transfers reveal that mutations in two proteins, a calcium-dependent protein kinase (PfCDPK5) and a P-type cation-ATPase (PfATP4), are necessary to impart full resistance to these compounds. A pyrazoleamide compound causes a rapid disruption of Na^+^ regulation in blood-stage *Plasmodium falciparum* parasites. Similar effect on Na^+^ homeostasis was recently reported for spiroindolones, which are antimalarials of a chemical class quite distinct from pyrazoleamides. Our results reveal that disruption of Na^+^ homeostasis in malaria parasites is a promising mode of antimalarial action mediated by at least two distinct chemical classes.

## Abstract (web summary)

Novel antimalarial drugs are urgently needed to combat parasite drug resistance. Here, Vaidya *et al*. describe a new chemical class of potent antimalarial compounds that act by disrupting the parasite's sodium homeostasis.

## Graphical abstract

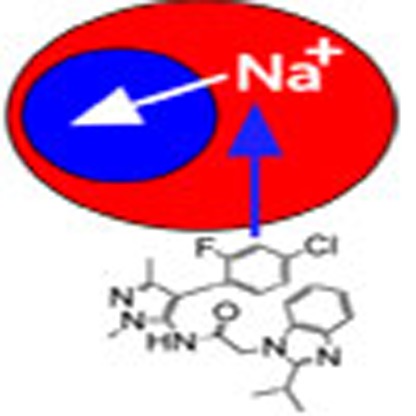

It is a well-established fact that microbial pathogens subjected to drug pressure tend to develop resistance to the drug. The probability of resistance emergence increases in proportion to the size of the population exposed to the antimicrobials. With hundreds of millions of malaria cases being treated with antimalarial drugs each year and with each individual patient bearing hundreds of billions of malaria parasites^1^, it is necessary to continue to feed the antimalarial pipeline with new drugs to counter the likely emergence of resistance. The emergence and spread of chloroquine-resistant *Plasmodium falciparum* has been a major contributing factor for the resurgence in malaria morbidity and mortality during the 1980s and 1990s (ref. 2), which only now seems to be abating with the advent of artemisinin combination therapy and other interventions^3^,^4^. Reports of delayed clinical response to artemisinin derivatives in Southeast Asia as a harbinger of resistance emergence^5^,^6^,^7^ provide further impetus for the need to have an available robust antimalarial pipeline. Over the past decade, the Medicines for Malaria Venture (MMV) has spearheaded efforts of academic and industrial partners to discover and develop antimalarial drugs. Several new compounds at various stages of development have been identified through these efforts^8^.

We describe here our investigations of a new chemical class of antimalarial compounds with highly potent activity against *P. falciparum* and *P. vivax*, the most prevalent species causing human malaria. These compounds are active against parasites resistant to currently used antimalarials and are also inhibitory to the onward development of the sexual stages of *P. falciparum* indicating their potential to be an effective means for treating malaria and for its transmission. Genetic and biochemical studies indicate that the pyrazoleamides are likely to affect a cation-pumping P-type ATPase, resulting in rapid disruption of Na^+^ homeostasis in intraerythrocytic *P. falciparum*. This mode of action is similar to a recent demonstration that NITD609 (ref. 9), an antimalarial spiroindolone under development with a very different chemical structure, also disrupts Na^+^ homeostasis in malaria parasites^10^.

## Results

### Pyrazoleamides as potent inhibitors of human *Plasmodium* spp

**Figure 1:**
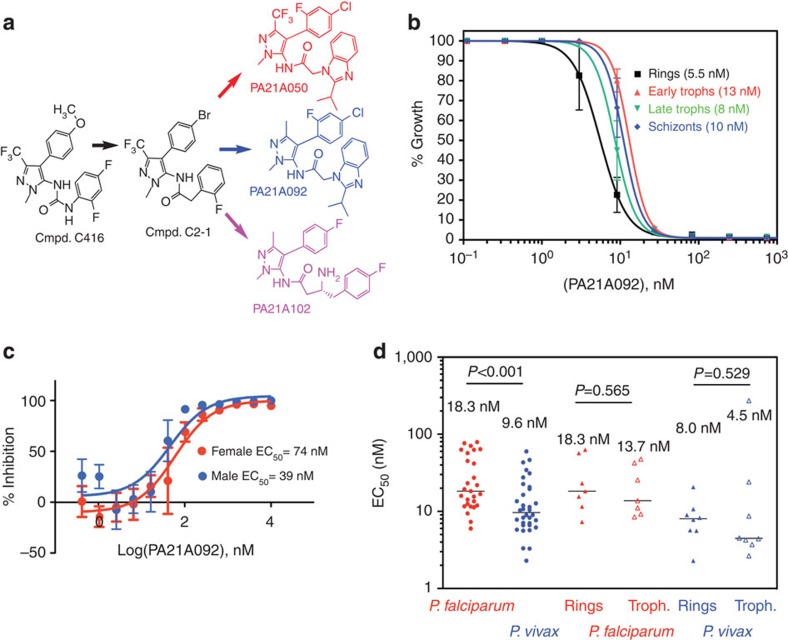
Structure and antimalarial activities of pyrazoleamide compounds. (**a**) Structures of the hit and lead compounds. (**b**) Growth inhibition assays of PA21A092 against the indicated stages of Dd2 line of *P. falciparum*. (**c**) PA21A092 inhibits male (blue) and female (red) gamete production by mature gametocytes. EC_50_ values are estimated to be 39 and 74 nM for male and female gamete production, respectively; methylene blue as a positive control had EC_50_ values of 39 and 43 nM, respectively, in these assays. (**d**) PA21A092 is active against clinical field isolates of *P. falciparum* (red circles) and *P. vivax* (blue circles) in *ex vivo* growth inhibition assays with equal potency against ring (closed triangles) and trophozoite (open triangles) stages in both species.

The initial hit compounds C416, a pyrazoleurea derivative, and C2-1, a pyrazoleamide derivative, (Fig. 1a) were identified through a structure-based *in silico* screening of a compound library^11^ and showed growth inhibitory activity with effective concentration for 50% growth inhibition (EC_50_) of 150 and 50 nM, respectively, against *P. falciparum*. An extensive medicinal chemistry campaign (to be described in detail elsewhere) was conducted by synthesizing variants of pyrazoleamide compound C2-1 and leading to a series of compounds with low nanomolar activity against *P. falciparum*. Structures of three of the late lead compounds, PA21A050 (EC_50_: 0.7 nM), PA21A092 (EC_50_: 5 nM) and PA21A102 (EC_50_: 8 nM), are shown in Fig. 1a. On the basis of its biological, pharmaceutical and toxicological profiles, compound PA21A092 was designated as a preclinical drug candidate to be developed for first-in-human studies. Relatively equal activity of PA21A092 was observed over a 48-h period against *P. falciparum*, regardless of the stage of parasites used in the assay, with EC_50_ values ranging from 5 to 13 nM (Fig. 1b). The functional viability of *P. falciparum* mature Stage V male and female gametocytes as manifested by their ability to form male and female gametes, respectively, was inhibited when exposed to PA21A092 with an EC_50_ of 39 and 74 nM, respectively (Fig. 1c), indicating its potential to act also as a transmission-blocking drug. A panel of eight *P. falciparum* lines resistant to a number of currently used antimalarial drugs were susceptible to compound 21A092 (Supplementary Table 1), suggesting a mode of action different from currently used antimalarials. Furthermore, PA21A092 was tested against clinical isolates of *P. falciparum* and *P. vivax* infecting patients living in an area with high prevalence of multiple drug resistance. Using an *ex vivo* assay^12^, we found that both species were highly susceptible to PA21A092, with a median EC_50_ of 18 nM against *P. falciparum* from 32 patients and 10 nM against *P. vivax* from 35 patients (Fig. 1d).

### *In vivo* efficacy of pyrazoleamides against *P. falciparum*

**Figure 2:**
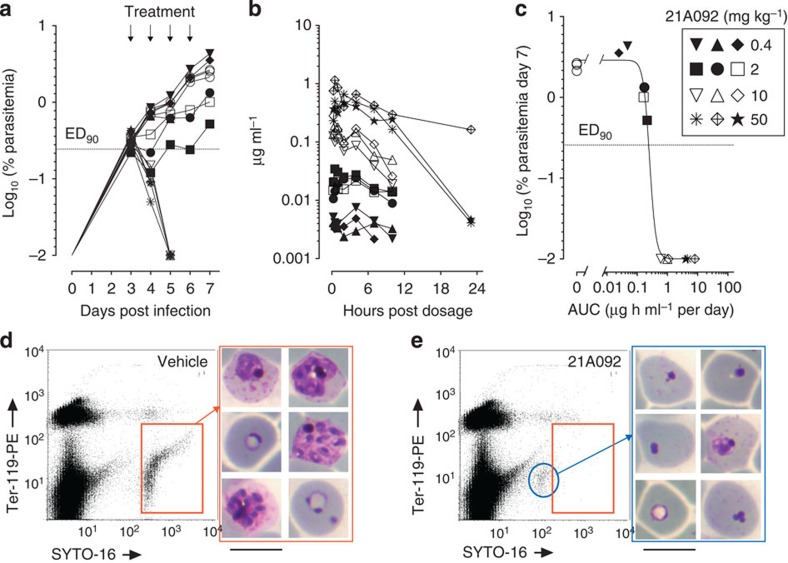
*In vivo* efficacy of PA21A092 against *P. falciparum*. Four indicated doses of PA21A092 were administered orally to groups of three NOD/scid/IL2Rγ^null^ mice each engrafted with human erythrocytes and infected with *P. falciparum*. The compound was administered starting on day 3 post infection for 4 consecutive days. Parasitemia was assessed each day from day 3 post infection up to 7 days (**a**). Concentrations of PA21A092 were measured by LC/MS in each mouse at 0.25, 0.5, 1, 2, 4, 7, 10 and 23 h after the first dose (**b**). The estimated drug exposure necessary to inhibit *P. falciparum* parasitemia on day 7 post infection by 90% (AUC_ED90_) was 0.24 μg h ml^−1^ per day^−1^ (**c**). Comparison of morphology of parasitized human RBC (as observed in Giemsa-stained thin blood smears prepared on day 7 after infection) in vehicle-treated (**d**) and PA21A092-treated (**e**) mice revealed normal stages of parasites in control but erythrocytes with only highly pyknotic staining nuclei fragments in treated mice (scale bars, 10 μm). In **a**–**c**, each symbol represents individual mouse with the dose of compound indicated in the inset on the right. Open circles in **a** are mice treated with the vehicle only.

We used nonobese/severe combined immunodeficiency/IL2Rγ^null^ (NOD/SCID/IL2Rγ^null^) mice engrafted with human erythrocytes and infected with an adapted line of *P. falciparum* as a model to assess *in vivo* efficacy of the pyrazoleamide compounds^13^,^14^. This model has been used previously for examining many different antimalarials for their *in vivo* activity against the human malaria parasite *P. falciparum*^15^. Groups of three mice each were treated once a day for 4 consecutive days, beginning on day 3 post infection, with four oral dose levels of PA21A092. As shown in Fig. 2a, PA21A092 was highly effective when administered orally with effective dose for 90% parasitemia reduction (ED_90_) of 2.5 mg kg^−1^. Compound PA21A050 had ED_90_ of 0.9 mg kg^−1^ (Supplementary Fig. 1), whereas PA21A102 had an ED_90_ of 4 mg kg^−1^ (Supplementary Fig. 2). The pharmacokinetics of PA21A092 in the mice employed in the therapeutic efficacy study was studied by taking 25 μl serial blood samples up to 23 h after the first dose. As shown in Fig. 2b and Supplementary Table 2, oral pharmacokinetics were approximately linear in the dose range used. Assuming no significant accumulation during treatment, the estimated drug exposure necessary to inhibit *P. falciparum* parasitemia on day 7 post infection by 90% with respect to control mice (area under the curve at 90% effective dose, AUC_ED90_) was 0.24 μg h ml^−1^ per day (Fig. 2c). In this *in vivo* assay, the AUC_ED90_ is the average daily exposure necessary to achieve no net parasite growth on day 7. The maximum parasite clearance was achieved at PA21A092 blood exposure of above 1 μg h ml^−1^ per day and was comparable, in this model, to that seen with artesunate, which is thus far the fastest-acting antimalarial in use. Erythrocytes with only remnants of parasite nuclei were seen following 2-day treatments with the pyrazole compounds (Fig. 2d,e).

### Investigating *P. falciparum* resistant to pyrazoleamides

The lack of cross-resistance to the pyrazoleamides in parasites resistant to currently used antimalarial drugs suggests a potentially novel mode of action for this new chemical class. To gain mechanistic insight into the action of the pyrazoleamides, we derived *P. falciparum* lines resistant to the initial hit compound C2-1. Our assessment of resistance frequency for compounds PA21A050 and PA21A092 indicated a rate of ≪3 × 10^−9^ under a standard protocol^16^ of continuous exposure to 10 × EC_50_ concentration of the compounds, suggesting a low propensity for parasite resistance development against these compounds. Therefore, resistant parasite lines were derived by exposure of the Dd2 line of *P. falciparum* to 3 × EC_50_ concentration of the compound C2-1. Three lines resistant to compound C2-1 with an EC_50_ of ~20-fold higher than the wild-type parasites were derived through this process of exposure to a reduced level of the compound (Supplementary Fig. 3). These resistant parasites were cross-resistant to all active pyrazoleamide compounds, including the late lead and candidate compounds (Supplementary Table 3), suggesting a common mode of action for the pyrazoleamide series.

**Figure 3:**
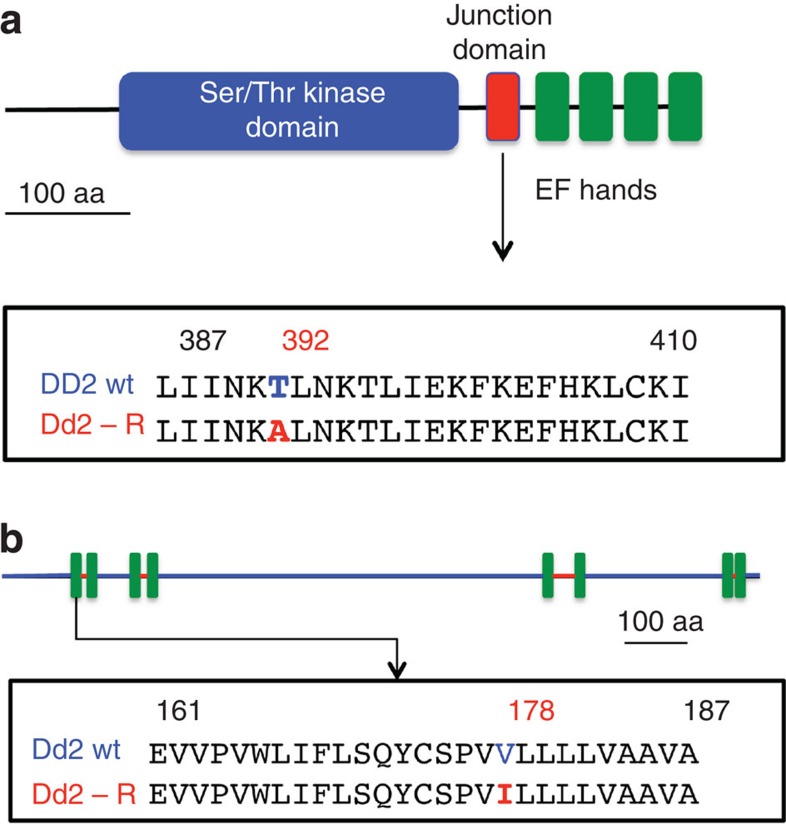
Mutations in compound C2-1-resistant parasites. A Thr to Ala mutation at position 392 in *PfCDPK5* is in its predicted junction domain (**a**), and a Val to Ile mutation at position 178 in *PfATP4* (**b**) is in the first predicted transmembrane domain.

To gain insight into the mode of action for and resistance to the pyrazoleamides, genomes of the Dd2 parental line and the three pyrazoleamide-resistant lines were sequenced using ‘next-generation’ sequencing technology^17^. Custom-designed bioinformatics for *P. falciparum* genome-sequence comparison^18^ revealed non-synonymous single-nucleotide polymorphism (SNP) in five genes: PF3D7_0411900, DNA polymerase alpha (mutation D133Y); PF3D7_0630900, DEAD/DEAH box ATP-dependent RNA helicase (N142K, a polymorphic site); PF3D7_1034300, thioredoxin-like associated protein 2, (K465E); PF3D7_1211900, non-SERCA calcium ATPase (V178I); and PF3D7_1337800, calcium-dependent protein kinase 5 (T392A). These mutations were common to all three resistant lines compared with the parental Dd2 parasites. An assessment of potential effects of these mutations as well as expression patterns of these genes led us to focus initial attention on two proteins: the calcium-dependent protein kinase 5 (PfCDPK5; PF3D7_1337800) and a P-type cation ATPase annotated as a non-SERCA calcium ATPase, PfATP4 (PF3D7_1211900). Dvorin *et al*.^19^ have shown PfCDPK5 to be essential for egress of progeny merozoites at the last step of erythrocytic schizogony by *P. falciparum*, and thus deemed an attractive drug target. The resistance-associated mutation, T392A, in PfCDPK5 was predicted to be in the junction region of the enzyme that links the kinase domain to the calmodulin-like EF-hand domains, and acts as a regulatory loop of the enzyme (Fig. 3a). PfATP4 was first described as a Ca^2+^ pump^20^,^21^ and recently found to bear mutations in *P. falciparum* parasites resistant to a new class of antimalarials, the spiroindolones^9^. Spillman *et al*.^10^ have provided evidence consistent with the hypothesis that PfATP4 is a P-type Na^+^ ATPase and a target for the spiroindolones. The resistance-associated mutation in PfATP4, a conservative V178I change, was localized to the predicted first transmembrane domain of the protein (Fig. 3b), and was different from the mutations observed by Rottmann *et al*.^9^ in spiroindolone-resistant parasites. The pyrazoleamide-resistant lines described here were not cross-resistant to a spiroindolone (Supplementary Fig. 3b).

### Mutations necessary for full resistance to pyrazoleamides

To assess the effects of expressing the mutated version of the enzyme (PfCDPK5^T392A^), a transgenic Dd2 line of *P. falciparum* was constructed in which the PfCDPK5^wt^ allele was replaced by PfCDPK5^T392A^ allele via single crossover allelic exchange recombination (Supplementary Fig. 4). When this transgenic parasite line (*Dd2::PfCDPK5*^*T392A*^) was examined for its response to pyrazoleamide compounds, it was found to have a slightly higher EC_50_ value compared with its parental line (Table 1 and Supplementary Fig. 5), showing that the *PfCDPK5*^*T392A*^ allele by itself appeared to result in a rather modest level of resistance to the pyrazoleamide compounds. We also derived transgenic Dd2 lines expressing either the *PfATP4*^*wt*^ or *PfATP4*^*V178I*^ allele from an ectopic site using a Mycobacteriophage recombination system^22^. Western blot analysis of transgenic parasites indicated relatively equal expression of the PfATP4 transgene (Supplementary Fig. 6). As shown in Table 1 and Supplementary Fig. 5, Dd2 parasites expressing *PfATP4*^*wt*^ responded to compound PA21A050 with an EC_50_ similar to the parental Dd2 line, whereas transgenic Dd2 line expressing *PfATP4*^*V178I*^ allele showed EC_50_ about twofold higher than the parental line. This indicated a slight gain of resistance by the transgenic parasites expressing *PfATP4*^*V178I*^ allele in a merodiploid state; however, the degree of resistance was much lower than that observed in the original resistant line. At this point, we used the *Dd2::PfCDPK5*^*T392A*^ line as the recipient of the wild-type or the mutant alleles of *PfATP4* through transfection. Whereas there was no change in the EC_50_ value for the compound PA21A050 in the *Dd2::PfCDPK5*^*T392A*^ line expressing *PfATP4*^*wt*^ allele, a resistance level similar to the original resistant line was observed in the *Dd2::PfCDPK5*^*T392A*^ line expressing the *PfATP4*^*V178I*^ allele (Table 1 and Supplementary Fig. 5). The transgenic parasites bearing double mutations were also resistant to several different pyrazoleamide compounds (Table 1 and Supplementary Fig. 5). These results strongly support the contention that a combined activity of mutated alleles of both *PfCDPK5* and *PfATP4* is necessary to impart full resistance to pyrazoleamide compounds in *P. falciparum*.

### A pyrazoleamide disrupts Na^+^ homeostasis in *P. falciparum*

**Figure 4:**
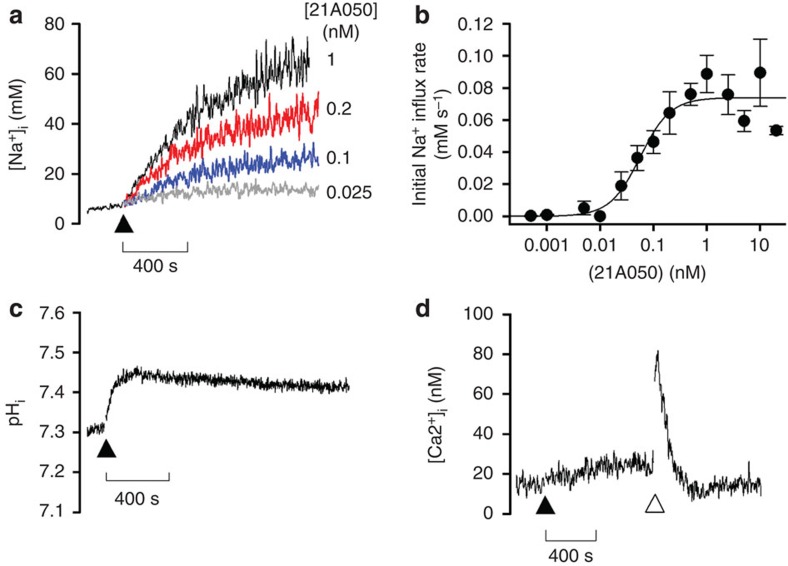
Rapid influx of Na^+^ in *P. falciparum* trophozoites following exposure to PA21A050. Intracellular concentrations of Na^+^, Ca^2+^ and pH in saponin-isolated parasites loaded with appropriate fluorescent probes were determined by ratiometric methods as described in ref. 10. In **a**,**c**,**d**, the pyrazoleamide was added at the time point indicated by the closed triangle. The traces and influx rates shown are, in each case, representative of those obtained from at least three independent cell preparations. In control experiments it was shown that there was no direct effect of the pyrazoleamide on the fluorescence signals of any of the three fluorescent ion indicators used (not shown). (**a**) Concentration-dependent increase in [Na^+^]_i_ in response to PA21A050. (**b**) Initial Na^+^ influx rate (±s.e.m.; calculated as described in ref. 10) plotted as function of [21A050]. The estimated IC_50_ for this effect was 0.08 nM. (**c**) PA21A050 at 1 nM caused a rapid rise in pH_i_ from 7.3 to 7.45, consistent with a lifting of the ‘acid-load’ proposed to be imposed by the action of the (H^+^ counter-transporting) Na^+^ ATPase^10^,^33^. (**d**) PA21A050, at 1 nM, had no significant effect on [Ca^2+^]_i_ in parasites suspended in an EGTA-buffered medium containing 1 μM free Ca^2+^. Addition of the endoplasmic reticulum Ca^2+^ pump inhibitor cyclopiazonic acid (unfilled triangle) resulted in a transient spike in [Ca^2+^]_i_.

As highlighted in a recent study^10^, PfATP4 bears a close resemblance to the closely related ENA (***e***xitus ***na***trum) Na^+^ ATPases that extrude Na^+^ ions from the cells of fungi and lower plants^23^. In the same study it was shown that antimalarial spiroindolones at therapeutic doses rapidly disrupt Na^+^ homeostasis in blood-stage *P. falciparum* parasites, and that spiroindolone-resistant parasites with mutations in PfATP4 (ref. 9) had reduced sensitivity to disruption of Na^+^ homeostasis by a spiroindolone^10^. As shown in Fig. 4a, the pyrazoleamide PA21A050 treatment of saponin-isolated parasites led to rapid increase in intracellular Na^+^ concentration ([Na^+^]_i_), doing so in a dose-dependent manner. Remarkably, a maximum initial Na^+^ influx rate of ~0.07 mM s^−1^ was reached with the addition of just 1 nM PA21A050 (Fig. 4b). The increase in [Na]_i_ was accompanied by an increase in intracellular pH (pH_i_, Fig. 4c), whereas there was no significant change in intracellular Ca^2+^ ([Ca^2+^]_i_) on addition of PA21A050 (Fig. 4d). These results are similar to those reported for the spiroindolones^10^ except that PA21A050 was more potent in its activity in this assay. Antimalarials such as artesunate and chloroquine have no effect on [Na^+^]_i_ in this assay^10^.

As *P. falciparum* matures from the ring stage to the trophozoite stage, new permeability pathways (also called *Plasmodial*-surface anion channels, or PSAC) are established in the erythrocyte membrane resulting in an increased permeability to a diverse range of low molecular weight solutes^24^,^25^. One consequence of this is an increase in [Na^+^] inside the erythrocyte cytosol^26^ and, hence, an inward [Na^+^] gradient across the plasma membrane of the intraerythrocytic trophozoite.

**Figure 5:**
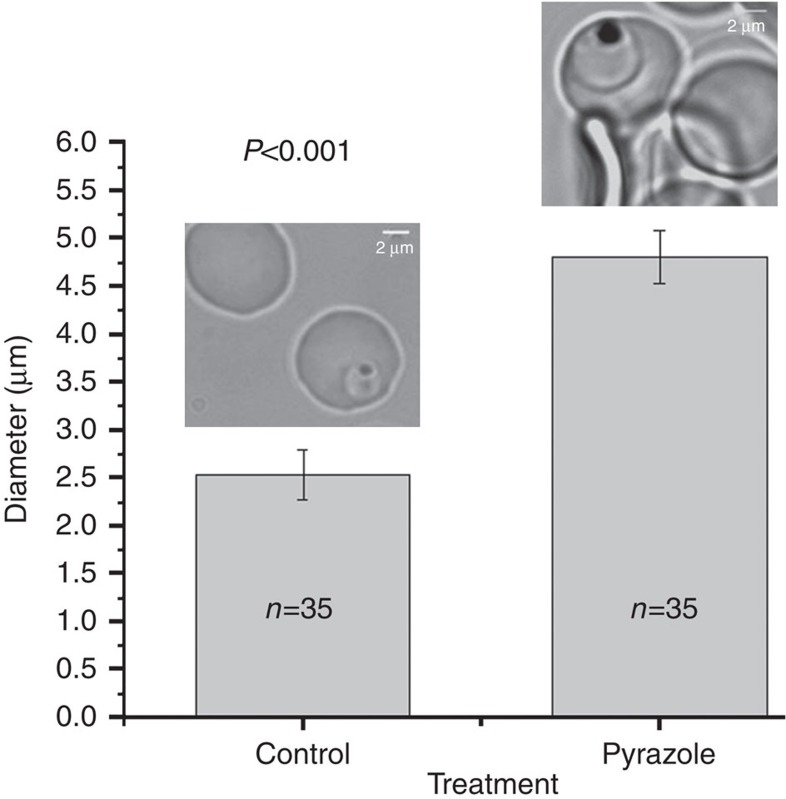
PA21A050 causes swelling of intraerythrocytic *P. falciparum*. Live cell images of trophozoite stages were obtained at 2 h from untreated and PA21A050-treated parasites attached to a glass-bottom Petri plate by a Nikon microscope equipped with an incubation chamber for regulating temperature and gas mixture. The diameter of the indicated number of parasites were measured from the images using the ImageJ software. Error bars indicate s.d.

We examined the effect of PA21A050 on *P. falciparum* development by time-lapse live cell imaging in erythrocytes starting from ring-stage parasites (Supplementary Movies 1 and 2). Ring-stage parasites progressed to develop into trophozoites while being exposed to PA21A050. At the late trophozoite stage there was visible increase in volume of the parasite, which was followed by dramatic apparent bursting; schizogony or merozoites were not observed (Supplementary Movie 2; notice swelling and bursting from 2,400 to 2,600 min time frames). These observations are consistent with the proposition that pyrazoleamides disrupt Na^+^ homeostasis, the effect of which becomes apparent coincident with the rise in [Na^+^] within the infected erythrocyte cytoplasm at the trophozoite stage. An increase in [Na^+^] in the parasite cytoplasm is predicted to give rise to an associated osmotic uptake of water and an associated swelling. To assess the extent of swelling, we measured the diameter of trophozoite-stage parasites in intact infected erythrocytes after 2 h of exposure to PA21A050 in still images of a number of parasites. As shown in Fig. 5, the average diameter of the treated trophozoite stage increased to 4.6 μm in 2 h compared with 2.5 μm in untreated control trophozoites from the same culture, consistent with the parasite having undergone significant swelling.

### Reduced sensitivity of parasites in low [Na^+^] medium

The results described above suggest that a contributing factor for antimalarial properties of these compounds could be toxicity associated with high [Na^+^]_i_. Recently, Pillai *et al*.^27^ have elegantly demonstrated that *P. falciparum* can tolerate a broad range of [Na^+^] in growth medium as long as appropriate osmotic conditions are maintained by KCl and sucrose. We took advantage of the low [Na^+^] growth medium used by Pillai *et al*.^27^ to ask whether parasites grown under low [Na^+^] condition had altered responses to antimalarial compounds that disrupt Na^+^ homeostasis. As shown in Table 2, Dd2 line of *P. falciparum* showed three- to fivefold reduced susceptibility to both the pyrazoleamide and spiroindolone compounds when grown in low [Na^+^] medium. It was interesting to note that the reduced susceptibility was more pronounced in a Dd2-21R line that was derived as resistant to a pyrazoleamide compound. These results further support the notion that Na^+^ homeostasis disruption is a key component of antimalarial action of these two chemically distinct classes of compounds.

## Discussion

We have described a multinational collaborative effort that has delivered a new chemical entity as a candidate antimalarial drug worthy of being developed for clinical investigations. Potent inhibitory activity of pyrazoleamides against multiple isolates of *P. falciparum* as well as *P. vivax* suggests the potential of deploying these compounds as part of a malaria control and elimination strategy. An added attractive feature of these compounds is their activity against the mature sexual stages of *P. falciparum*, a property not common to most of the currently used antimalarial drugs. Inhibition of gamete production by these compounds would prevent parasite mating and transmission by mosquitoes, a goal that has to be part of a malaria elimination programme. Results from *in vivo* efficacy studies described here demonstrate rapid clearance of *P. falciparum* when exposed to orally administered pyrazoleamides. This rate of clearance is reminiscent of clearance by artemisinin derivatives, which are the fastest-acting antimalarials in clinical use.

We provide evidence suggesting rapid disruption of Na^+^ homeostasis in intraerythrocytic parasites as a consequence of exposure to pyrazoleamides at pharmacologically relevant concentrations. It has been shown previously that the induction by the parasite of new permeability pathway/*Plasmodial*-surface anion channel in erythrocyte plasma membrane occurs as the ring-stage parasite transitions to the metabolically more active trophozoite stage^24^,^25^. As a consequence, [Na^+^] inside the erythrocyte cytoplasm increases^24^,^25^, yet [Na^+^] inside the parasite remains physiologically low. Spillman *et al*.^10^ have suggested that the maintenance of low [Na^+^]_i_ inside the parasite is mediated by a P-type Na^+^ ATPase pump encoded by the parasite, and that the new antimalarial spiroindolone, NITD246, interferes with the activity of this pump leading to a rapid net uptake of Na^+^. We show here that a pyrazoleamide has a similar effect to the spiroindolones on Na^+^ influx in saponin-isolated parasites. Furthermore, we show that just a 2-h exposure to a pyrazoleamide results in a significant increase in the volume of intact intraerythrocytic trophozoite-stage parasites, followed by a dramatic bursting of the parasite. These results, in combination with our observation of reduced efficacy of the pyrazoleamide and spiroindolone against *P. falciparum* parasites growing under low [Na^+^] conditions, provide strong evidence that these two chemically distinct classes of compounds share a common mode of action through disruption of Na^+^ homeostasis.

The finding that mutations in *PfATP4* are associated with acquisition of resistance to spiroindolone and pyrazoleamide compounds is consistent with the hypothesis that this P-type ATPase is a direct target of these compounds. However, some caution is warranted. A mutation in *PfCDPK5*, which by itself imparts minimal resistance to pyrazoleamides, greatly enhances the level of resistance in combination with mutated *PfATP4*. *PfCDPK5* has been shown to be essential in the very last step of schizogony before merozoites egress^19^. It has also been shown that mature schizont-stage parasites undergo swelling just before merozoite egress, and this is thought to facilitate the egress process^28^. The finding in this study of an association between *PfATP4* and *PfCDPK5* raises the possibility that the swelling is the result of increased Na^+^ influx, perhaps because of inhibition of PfATP4 activity, resulting from a cascade of events in which PfCDPK5 plays a regulatory function. Under this scenario, it might be envisioned that pyrazoleamides (and possibly spiroindolones) act as premature signals to initiate an egress-like cascade resulting in Na^+^ influx and swelling.

Further experiments are needed to understand details of events that lead to the demise of the parasites exposed to these new antimalarials. It is clear, however, that observations reported here are hinting at a hitherto unknown pathway in malaria parasites, which can be interfered with by at least two different classes of chemicals. Recent observations from our and other laboratories suggest additional compounds of unrelated chemical structures may also target this pathway. We look forward to unravelling mechanistic details of this pathway with a view to discovering other new antimalarial drugs.

## Methods

### Synthesis schemes for pyrazoleamides

**Figure 6:**
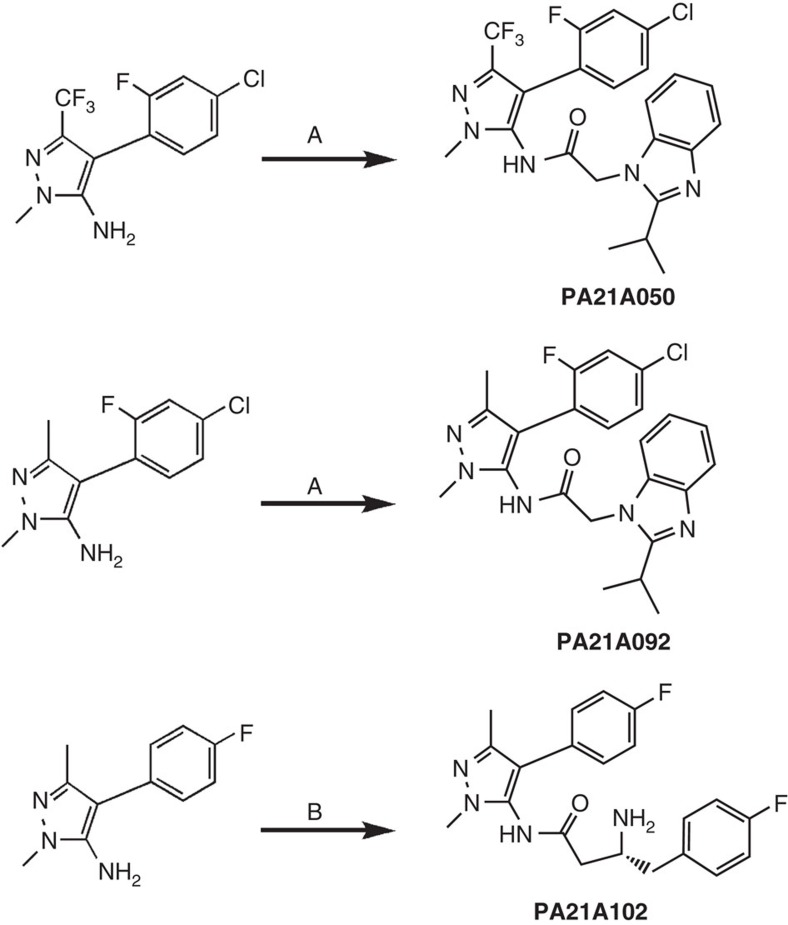
Synthetic schemes and reaction conditions for pyrazoleamides. (**a**) For PA21A050 and PA21A092: 2-(2-isopropyl-1H-benzo[d]imidazol-1-yl)acetic acid, Mukaiyama’s reagent (2-chloro-1-methylpyridinium iodide), triethylamine, in dichloromethane/tetrahydrofuran, microwave, 75 °C, 30 min. (**b**) For PA21A102: (i) Fmoc-(R)-3-amino-4-(4-fluorophenyl)butanoyl chloride in dichloromethane, (ii) DBU (1,8-diazabicyclo[5.4.0]undec-7-ene) in ethyl acetate.

All reagents and starting materials described in the procedures are commercially available and were used without further purification. Synthetic route for N-(4-(4-chloro-2-fluorophenyl)-3-(trifluoromethyl)-1-methyl-1H-pyrazol-5-yl)-2-(2-isopropyl-1H-benzo[d]imidazol-1-yl)acetamide (Compound PA21A050) is illustrated in Fig. 6. To a mixture of 2-(2-isopropyl-1H-benzo[d]imidazol-1-yl)acetic acid (0.16 mmol) and Mukaiyama’s reagent (2-chloro-1-methylpyridinium iodide, 0.38 mmol) in 1.5 ml anhydrous dichloromethane (DCM), 4-(4-chloro-2-fluorophenyl)-3-(trifluoromethyl)-1-methyl-1H-pyrazol-5-amine (0.12 mmol), triethylamine (0.40 mmol) and 0.5 ml of anhydrous tetrahydrofuran were added. The mixture was vortexed and subjected to microwave irradiation for 30 min at 75 °C to give a deep-green clear solution. Then 80 ml of ethyl acetate was added and the solution was washed with 80 ml of saturated NaHCO_3_ twice, and with brine, and then dried over MgSO_4_. After solvent removal and purification on a flash silica gel column using ethyl acetate and hexane as eluent, a slightly brown-coloured solid was obtained. After recrystallization in ethyl acetate/hexane, 45 mg of Compound PA21A050 was obtained as a white powder. Yield: 76%. Purity: >95% by HPLC (UV at 220 nm). ^1^H NMR (500 MHz, MeOD) *δ* 7.81–7.83 (m, 1H), 7.60–7.70 (m, 3H), 7.26–7.36 (m, 3H), 5.54 (s, 2H), 3.92 (s, 3H), 3.50–3.55 (m, 1H), 1.50 (d, *J*=6.9 Hz, 6H). ^13^C-NMR (126 MHz, MeOD) *δ* 169.30, 162.35, 162.02, 160.36, 142.93, 136.64, 136.55, 136.24, 134.19, 125.84, 123.87, 123.59, 119.31, 117.68, 117.60, 117.56, 117.39, 110.42, 46.39, 37.06, 27.47, 21.81. high-resolution mass spectrometry, electrospray ionisation (HRMS(ESI)): *M/Z* calculated for C_23_H_20_ClF_4_N_5_O+H^+^ [M+H^+^]: 494.1365. Found: 494.1358. Traces of liquid chromatography/mass spectrometry (LC/MS), ^1^H NMR and ^13^C-NMR of PA21A050 are shown in Supplementary Figs 7 and 8.

Synthetic route for N-(4-(4-chloro-2-fluorophenyl)-1,3-dimethyl-1H-pyrazol-5-yl)-2-(2-isopropyl-1H-benzo[d]imidazol-1-yl)acetamide (Compound **PA21A092)** is illustrated in Fig. 6. The synthesis followed the route for PA21A050 described above using 4-(4-chloro-2-fluorophenyl)-1,3-dimethyl-1H-pyrazol-5-amine (0.12 mmol) as starting material. After silica gel chromatography using ethyl acetate and hexane as eluent, 42 mg of Compound PA21A092 was obtained as a white powder. Yield: 80%, purity: >95% by HPLC (UV at 220 nm). ^1^H NMR (500 MHz, MeOD) *δ* 7.62–7.64 (m, 1H), 7.23–7.33 (m, 6H), 5.15 (s, 2H), 3.73 (s, 3H), 3.18–3.21 (m, 1H), 2.17 (s, 3H), 1.39 (d, *J*=6.8 Hz, 6H). ^13^C-NMR (126 MHz, MeOD) *δ* 169.25, 162.16, 162.04, 160.18, 147.16, 142.89, 136.24, 134.90, 133.76, 125.92, 123.80, 123.56, 119.25, 117.67, 117.46, 110.51, 110.31, 46.39, 35.79, 27.46, 21.82 and 12.67. HRMS (ESI): *M/Z* calculated for C_23_H_23_ClFN_5_O+H^+^ [M+H^+^]: 440.1648. Found: 440.1641. Traces of LC/MS, ^1^H NMR and ^13^C-NMR of PA21A092 are shown in Supplementary Figs 9 and 10.

Synthetic route for (*R*)-3-amino-4-(4-fluorophenyl)-N-(4-(4-fluorophenyl)-1,3-dimethyl-1H-pyrazol-5-yl)butanamide (Compound **PA21A102)** is illustrated in Fig. 6. To a solution of Fmoc-(*R*)-3-amino-4-(4-fluorophenyl)butanoyl chloride (43 mg, 0.20 mmol) produced from Fmoc-(*R*)-3-amino-4-(4-fluorophenyl)butanoic acid and thionyl chloride in 10 ml of anhydrous DCM were slowly added 4-(4-fluorophenyl)-1,3-dimethyl-1H-pyrazol-5-amine (31 mg, 0.15 mmol) in 5 ml of anhydrous DCM. The reaction mixture was stirred at room temperature overnight. The reaction mixture was quenched with methanol and solvents were removed. The residue was purified via silica gel with MeOH/DCM to obtain Fmoc-protected product. Fmoc-protected product was dissolved in 10 ml of ethyl acetate and 0.20 mmol of 1,8-diazabicyclo[5.4.0]undec-7-ene) in ethyl acetate was added. After 20 min, 20 ml of ethyl acetate was added and the mixture was washed with 20 ml of water. The organic layer was collected and solvent was removed. The residue was dissolved in MeOH and acidified with 0.2 N HCl. The solution was purified via preparatory RP-HPLC, eluting with H_2_O/CH_3_CN gradient (+0.05% trifluoroacetic acid, TFA). Product fractions were collected and concentrated. The residue was dissolved in a small amount of 2 M HCl in methanol and, after concentration *in vacuo*, 48 mg of Compound PA21A102 was obtained as an HCl salt. Yield: 76%. Purity: >95% by HPLC (UV at 220 nm). To prepare the compound as free base, the salt form was dissolved in saturated Na_2_CO_3_ solution and extracted with ethyl acetate followed by removal of solvent. ^1^H NMR of free base form (500 MHz, MeOD) *δ* 7.30–7.32 (m, 2H), 7.20–7.22 (m, 2H), 7.11–7.15 (m, 2H), 7.03–7.07 (m, 2H), 3.68 (s, 3H), 3.38–3.44 (m, 1H), 2.72–2.76 (m, 1H), 2.62–2.66 (m, 1H), 2.51–2.55 (m, 1H), 2.37–2.42 (m, 1H), 2.24 (s, 3H). ^13^C-NMR (126 MHz, MeOD) *δ* 174.60, 164.17, 162.23, 146.13, 135.61, 135.58, 134.56, 132.06, 131.99, 131.90, 131.84, 129.70, 116.44, 116.31, 116.27, 116.14, 51.07, 43.28, 42.56, 35.57 and 12.78. HRMS (ESI): *M/Z* calculated for C_21_H_22_F_2_N_4_O+H^+^ [M+H^+^]: 385.1834. Found: 385.1832. Traces of LC/MS, ^1^H NMR and ^13^C-NMR of PA21A102 are shown in Supplementary Figs 11 and 12.

### Parasites and growth inhibition assays

*P. falciparum* lines (obtained from Malaria Research and Reference Reagent Resource Center, http://www.mr4.org) were cultured under standard conditions in RPMI1640 medium supplemented with 0.5% Albumax in 90% N_2_, 5% O_2_ and 5% CO_2_. Parasite growth inhibition was assessed by a modified version of the method originally described in ref. 29. The method assessed parasite growth as reflected by incorporation of ^3^H-hypoxanthine by parasites. *P. falciparum* parasites in culture were exposed to graded dilutions of test compounds for 48 h and incorporation of ^3^H-hypoxanthine over the last 24 h into parasite nucleic acids was determined by liquid scintillation spectroscopy. The dose–response data were analysed using nonlinear regression analysis (Prism GraphPad), and the EC_50_ was derived using an inhibitory sigmoid maximum effect (*E*_max_) model. For assessing parasite growth inhibition under low [Na^+^] conditions, we used the medium composition designated 4suc:6 KCl as described in ref. 27. Briefly, the medium contained all ingredients specified for RPMI16040 except that NaCl, NaHCO_3_ and Na_2_HPO_4_ were replaced by 64.8 mM KCl, 28.6 mM KCO_3_ and 5.64 mM K_2_HPO_4_. In addition, 84.3 mM sucrose was included in the medium. Addition of 10% human serum to the 4suc:6 KCl medium was estimated to result in ~7 mM Na^+^ concentration in this medium. Efficacy of compounds to inhibit parasite growth in the low [Na^+^] 4suc:6 KCl+10% human serum medium was assessed in parallel with that in standard RPMI1640 medium by ^3^H-hypoxanthine incorporation as described above.

### Dual *P. falciparum* male and female gamete formation assays

A recently reported assay was used to assess effects of various doses of PA21A092 on gamete production by the NF54 line of *P. falciparum*^30^. *P. falciparum* NF54 strain gametocytes were produced by standard methods. Briefly, asexual parasites in the log phase of growth were passaged to 1% parasitemia/4% haematocrit and cultured in RPMI medium+25 mM HEPES+50 mg l^−1^ hypoxanthine+2 g l^−1^ sodium bicarbonate+10% human serum with daily medium changes while maintaining 37 °C and 3% O_2_/5% CO_2_/92% N_2_ at all times. Under these conditions, gamete formation is maximal by 14 days in culture and the assay performed. Cultures were divided into 200-μl microcultures containing test compound dilutions with no more than 0.5% dimethylsulphoxide. After 24 h incubation with the test compounds, gamete formation was stimulated by addition of 2.5 μM xanthurenic acid. At 20 min, exflagellation was recorded by time-lapse microscopy using × 10 objective and quantified using a custom algorithm. At 24 h post addition of xanthurenic acid, a Cy3-labelled anti-Pf25 antibody was added at 1:2,000 dilution to the cultures that specifically stains female gametes that were then recorded by fluorescence microscope using a × 20 objective and quantified using a custom algorithm.

Percent inhibition of male or female gamete formation was calculated with respect to the positive (10 μM methylene blue) and negative (DMSO) controls and the whole assay was repeated four times using independent cultures.

### Assessing *in vivo* efficacy

Efficacy of pyrazoleamides was examined in 10- to 12-week-old female NOD/scid/IL2R_γ_^null^ mice engrafted with human erythrocytes and infected with an adapted *P. falciparum* line as described previously^13^,^14^. Briefly, three mice per group were inoculated intravenously with 2 × 10^7^ parasitized erythrocytes (*P. falciparum* strain 3D7^0087/N9^ adapted for *in vivo* growth). Beginning from day 3 after the inoculation, mice were administered the stated doses of the compound orally for 4 consecutive days. Chloroquine at 2.5, 5 and 10 mg kg^−1^ was used as a positive control. A group of infected mice given oral dosing of the vehicle serves as the control. Parasitemia was measured from days 3 to 7 after infection by flow cytometry, counting 10^6^ events. SYTO-16 was used for staining parasite nucleic acids, and non-cytolytic antimouse erythrocyte monoclonal antibody Ter-119 conjugated with phycoerythrine was used for differentiating mouse and human erythrocytes. Thin smears of blood stained with Giemsa were examined and representative infected erythrocytes photographed on days 5 and 7 after infection in each mouse. Animal experiments were performed at the Association for Assessment and Accreditation of Laboratory Animal Care International-accredited GlaxoSmithKline Laboratory Animal Science facility in Tres Cantos (Madrid, Spain). All the experiments were approved by the GlaxoSmithKline Diseases of the Developing World Group Ethical Committee.

### Pharmacokinetics of PA21A092 in NOD/SCID/IL2Rγ^null^ mice

The levels of PA21A092 in blood upon oral administration were measured for all the mice and dosing levels included in the efficacy study. Serial samples of peripheral blood (25 μl) were taken at 0.25, 0.5, 1, 2, 4, 7, 10 and 23 h after the first administration of PA21A092, mixed with 25 μl of water for erythrocyte lysis and immediately frozen and stored at −80 °C until analysis. Protein precipitation by liquid–liquid extraction was performed in a 96-well plate with filter system (MultiScreen Solvinert 0.45 μm Hydrophobic PTFE; Millipore). Briefly, 120 μl of AcN:MeOH (80:20; v-v) containing internal standard were added to 10 μl of blood/saponin lysate sample per well. The plates were vortexed for 10 min, filtrated and the filtrates were analysed using LC-MS/MS in positive ion mode with electrospray (Sciex API 4000 Triple Quadrupole Mass Spectrometer, Sciex, Division of MDS Inc., Toronto, Canada). Noncompartmental analysis was performed using Phoenix, version 6.3. (Phoenix WinNonlin Copyright 1998–2012, Certara L.P.) and the main pharmacokinetic parameters were estimated. Additional statistical analysis of the data was performed with GraphPad Prism Version 5.01 (GraphPad Software Inc., San Diego CA).

### Testing in field isolates of *P. falciparum* and *P. vivax*

*Plasmodium spp.* isolates were collected from patients with malaria attending outpatient clinics in Timika (Papua Province, Indonesia), a region highly prevalent for multidrug-resistant strains of *P. vivax* and *P. falciparum*. Patients with symptomatic malaria were recruited into the study if singly infected with *P. falciparum* or *P. vivax* and a parasitaemia of between 2,000 and 80,000 μl^−1^. After written informed consent was obtained, venous blood (5 ml) was collected by venepuncture. Ethical approval for this project was obtained from the Human Research Ethics Committee of the Northern Territory Department of Health & Families and Menzies School of Health Research (HREC 2010-1396), Darwin (Australia) and the Eijkman Institute Research Ethics Commission (EIREC-47), Jakarta (Indonesia). *Plasmodium* drug susceptibility was measured using a protocol modified from the World Health Organization microtest as described previously^12^. Two hundred microlitres of a 2% haematocrit blood medium mixture, consisting of RPMI1640 medium plus 10% AB^+^ human serum (*P. falciparum*) or McCoy’s 5A medium plus 20% AB^+^ human serum (*P. vivax*), were added to each well of predosed drug plates containing 11 serial concentrations (twofold dilutions) of the compound PA21A092. A candle jar was used to mature the parasites at 37.0 °C for 32–56 h. Incubation was stopped when >40% of ring-stage parasites had reached the mature schizont stage (that is, four or more distinct nuclei per parasite) in the drug-free control well.

Thick blood films made from each well were stained with 5% Giemsa solution for 30 min and examined microscopically. The number of schizonts per 200 asexual stage parasites was determined for each drug concentration and normalized to that of the control well. The dose–response data were analysed using nonlinear regression analysis (WinNonLn 4.1; Pharsight Corporation), and the EC_50_ was derived using an *E*_max_ model.

### Derivation of resistant parasites

*P. falciparum* line Dd2 was used to derive parasite lines resistant to the initial hit pyrazoleamide compound C2-1. Three flasks each containing 10^8^ parasitized erythrocytes were continuously exposed to 3 × EC_50_ concentration of the compound with medium changes each day and fresh provision of human red blood cell (RBC) once a week. Emergence of parasites was monitored by examining stained thin smears. Each of the flasks yielded resistant parasites, which were confirmed to be resistant to pyrazoleamides.

### Whole-genome sequencing and analysis

Parental Dd2 *P. falciparum* line and the three pyrazoleamide-resistant lines were sequenced using the Illumina GAII and the amplification-free methodology^17^. From each clone, 300-bp fragments of genomic DNA were prepared by focused sonication. An individual sequencing lane was used to produce 33–40 million paired 76-base reads for each sample. Between 55 and 71% of sequencing reads for each sample mapped uniquely to the *P. falciparum* clone 3D7 reference genome, with read-spacing accurately reflecting the expected fragment size.

Dd2 reads were aligned to the 3D7 genome and any single-base differences or small indels were identified by iCORN^18^ to iteratively change the 3D7 sequence into the one that more closely resembled the actual sequence for Dd2 (hereafter termed Dd2′). Only sequence differences within regions of 10–15 × read coverage were accepted to minimize the possibility of using incorrectly mapped sequences, and iCORN was run for 17 iterations (until no further sequence differences could be found between the gene sequences of 3D7 and Dd2). In all, 41,841 base substitutions were made and 16,638 indels corrected.

Reads from each derived line were mapped to the Dd2′ reference to identify SNPs and indels, using three iterations of iCORN so that SNPs or indel could be identified even within polymorphic loci. Sequence variants were only identified within regions of at least × 40 coverage on Dd2′. Using a custom Perl script, genes were identified where a variant was found within the reads for all three derived clones. Five SNPs were found at the same base position in each of the three derived clones. These were the only differences observed when compared with Dd2′.

### Approaches to generate transgenic parasites

A DNA fragment corresponding to nucleotides 9–1,704 of the coding region of *PfCDPK5* (PlasmoDB ID PF3D7_1337800) and having a 5′-*SpeI* site and an in-frame BsiWI site was amplified using Vent DNA polymerase (New England Biolabs) and cloned into pCR-Blunt (Invitrogen). The DNA sequence of the amplified fragment was confirmed. The *PfCDPK*^*5T392A*^ mutation was introduced into the plasmid using the QuikChange Site-Directed Mutagenesis Kit (Stratagene). The resulting SpeI-BsiWI DNA fragments were cloned into the *P. falciparum* vector pCC1-3HA and used to transform parasite strain Dd2. WR99210-resistant parasites were obtained, subjected to one drug-off/drug-on cycle to select for transgene insertion. The site-specific integration was confirmed with PCR using both gene-specific and integration-specific primers, as illustrated in Supplementary Fig. 4. The primers correspond to the following DNA regions: 5′-primer (−425 to −401 nucleotides upstream of the ATG codon); 3′-primer (+96 to +123 nucleotides downstream of the Stop codon); C primer (nucleotides 1,671–1,704 of the coding sequence); 3HA primer corresponds to the influenza virus hemagglutination antigen epitope.

Full-length *PfATP4* gene was amplified by PCR using genomic DNA from either the wild-type Dd2 parasite (to produce *PfATP4*^*wt*^), or Dd2-R21 parasites resistant to compound C2-1 (to produce *PfATP4*^*T392A*^). DNA sequences were confirmed and the genes were cloned into the pLN plasmid^22^ to be expressed under the calmodulin promoter^22^. Transfections of *P. falciparum* parasites were carried out as described elsewhere^31^,^32^. Briefly, ring-stage parasites at 5% parasitemia were electroporated with 50 μg plasmid DNA isolated using the Qiagen Plasmid Maxi Kit. Electroporation was conducted using a Bio-Rad GenePulser set at 0.31 kV and 960 μF. Two aliquots of DNA were electroporated for each transgene. Transfected parasites were maintained under drug pressure: 5 nM WR99210 for hdhfr (human dihydrofolate reductase), 2.5 μg ml^−1^ blasticidin for blasticidin deaminase and 125 μg ml^−1^ G418 for the neomycin selectable markers.

The following transgenic parasites were used in this study:

- *Dd2::PfCDPK5*^*T392A*^—The Dd2 line with *PfCDPK5* allele bearing T392A mutation seen in Dd2-21R-resistant line. The wild-type allele is replaced with the mutated allele that also bears a 3 × HA tag at the C terminus.
- *Dd2attB+PfATP4*^*wt*^–The Dd2attB line with the wild-type *PfATP4* gene inserted at the attB site. This line is a merodiploid with two copies of the wild-type *PfATP4*.
- *Dd2attB+PfATP6*^*V178I*^—The Dd2attB line with PfATP4 bearing V178I mutation inserted at the attB site. This line is a meroheterozygote expressing both the wild-type and V178I alleles of *PfATP4*.
- *Dd2::PfCDPK5*^*T392A*^*+PfATP4*^*wt*^—Transgenic Dd2 line bearing T32A mutation in its *PfCDPK5* gene transfected with the same plasmid used to generate *Dd2attB+PfATP4*^*wt*^ line. The plasmid replicates as an episome.
- *Dd2::PfCDPK5*^*T392A*^*+PfATP4*^*V178I*^—Transgenic Dd2 line bearing T392A mutation in its *PfCDPK5* gene transfected with the same plasmid used to generate the *Dd2attB+PfATP4*^*V178I*^ line. The plasmid replicates as an episome.

### Spectrofluorometer measurements of [Na^+^]_i_, [Ca^2+^]_i_ and pH_i_

Trophozoite-stage, 3D7 *P. falciparum* parasites (36–40 h post invasion) were functionally isolated from their host erythrocytes by treatment with saponin (0.05% w/v, equating to 0.005% w/v of the active agent sapogenin) and then loaded with the appropriate fluorescent probe (Sodium-binding BenzoFuran Isophthalate for [Na^+^]_i_; BCECF-AM, 2′,7′-bis-(2-carboxyethyl)-5,6-carboxyfluorescein, acetoxymethyl ester for pH_i_; Fura-2, 2-(6-(bis(2-((acetyloxy)methoxy)-2-oxoethyl)amino)-5-(2-(bis(2-((acetyloxy)methoxy)-2-oxoethyl)amino)-5-methylphenoxy)ethoxy)-2-benzofuranyl) for [Ca^2+^]_i_) and suspended in a medium containing 125 mM NaCl, 5 mM KCl, 25 mM HEPES, 20 mM glucose and 1 mM MgCl_2_; pH 7.10. For the Fura-2/[Ca^2+^]_i_ experiments, this solution was supplemented with 1 mM EGTA and sufficient CaCl_2_ to result in a final free extracellular Ca^2+^ concentration of 1 μM. Detailed methods for dye-loading, cell concentrations, calibration, rate calculations and appropriate ratiometric measurement using a spectrofluorometer were as described in ref. 10. Calibrations were performed for each experiment and are shown in Supplementary Fig. 13 along with the equations used for assessing ion concentrations.

### Live cell imaging

*P. falciparum* line 3D7 parasites were synchronized through osmotic lysis of late-stage parasites incubated with 500 mM alanine. Parasitized erythrocytes in phenol red-free medium were attached to a glass-bottom Petri dish coated with concanavalin A. Images were obtained in an inverted Nikon Eclipse microscope equipped with a chamber stage in which temperature (37 °C) and gas mixture (5% O_2_, 5% CO_2_ and 95% N_2_) were maintained. For time-lapse movies, images were collected every 30 min for 45 h using autofocus setting by a × 100 objective lens in phase contrast. Images were processed and assembled into videos using the ImageJ software. Static phase-contrast images of trophozoite-stage parasites either untreated or treated with 10 nM PA21A050 were obtained after 2-h incubation under the inverted Nikon microscope. The ImageJ software was used to measure diameters of untreated and treated parasite images assuming the erythrocyte diameter to be 8 μm.

## Author contributions

A.B.V. designed and directed biological studies, provided input into medicinal chemistry, analysed the data and wrote the manuscript; J.M.M. carried out parasitological studies, derived resistant parasites, carried out transfections and generated genetic data; Z.Z. carried out organic synthesis of compounds and conducted their chemical characterization with assistance from M.S.; S.D. carried out live cell imaging and videography; T.M.D. assisted in characterization of transfected parasites; T.D.O. and M.B. carried out whole-genome sequencing and bioinformatics; N.J.S. and K.K. designed and conducted ion homeostasis experiments, and wrote parts of the manuscript; M.W., P.S., S.A.C., A.C., A.N. and J.B. provided input into medicinal chemistry and pharmacology of compounds; J.M., G.W., B.F.S. and R.N.P. conducted and analysed *in vitro* tests on field isolates; I.A.-B., S.F., M.B.J.-D., M.S.M. and F.J.G. designed, directed, conducted and analysed all experiments involving the NOD/scid/IL2R_γ_^null^ mice; V.M.A., A.R. and M.D. designed, conducted and analysed experiments involving sexual stages of parasites; S.K. provided input into medicinal chemistry and molecular dynamics; E.F. directed organic synthesis of compounds and provided input into medicinal chemistry and characterization of compounds; and L.W.B. carried out construction of transfection plasmids and assisted with analysis of genetic data.

## Additional information

**How to cite this article**: Vaidya, A. B. *et al*. Pyrazoleamide compounds are potent antimalarials that target Na^+^ homeostasis in intraerythrocytic *Plasmodium falciparum*. *Nat. Commun.* 5:5521 doi: 10.1038/ncomms6521 (2014).

**Accession codes:** Raw sequence reads from the whole-genome sequencing are deposited in the European Nucleotide Archive under study ERP000325 ( http://www.ebi.ac.uk/ena/data/view/ERP000325) with sample ID numbers ERS005005 for the Dd2 parent, and ERS005006, ERS005007 and ERS005008 for the three resistant clones.

## Supplementary Material

Supplementary Information Supplementary Figures 1-13, Supplementary Tables 1-3 and Supplementary References

Supplementary Movie 1 Three different untreated parasitized RBC visualized by time-lapse live videography. The first parasite was imaged for 1050 minutes, starting from early schizont stage that results in the rupture of schizont at 720 min mark followed by formation of a new ring stage parasite with dynamic morphology up to 1050 minute. This is followed by imaging of a parasite from ring to trophozoite stage over 1500 min. The last set of frames consists of 1470 min of imaging with schizont rupture seen at 1260 min mark.

Supplementary Movie 1 Live videography of parasitized RBC exposed to 10 nM PA21A050. This video depicts two parasites continuously imaged for 2700 min. There appears to be some progression of the parasites but also apparent swelling. Dramatic morphological changes followed by the bursting of the parasites are apparent between 1500 to 1800 min for the parasite at 4 o'clock position, and between 2400 to 2600 min for the parasite at 11 o'clock position.

## Figures and Tables

**Table 1:** Efficacy of pyrazoleamides and a spiroindolone in transgenic *P. falciparum*.

**Parasite line**	***In vitro*** **EC**_**50**_**, nM (±s.e.m.)**			
	PA21A050	PA21A092	**PA27-X41**	**NITD246**
Dd2	0.7 (±0.2)	12.9 (±2.0)	0.7 (±0.15)	0.15 (±0.01)
Dd2-R21	16 (±0.5)	133 (±8.0)	122 (±10)	0.21 (±0.04)
Dd2::PfCDPK5^T392A^	1.5 (±0.1)	23 (±3.0)	ND	0.17 (±0.04)
Dd2attB+PfATP4^wt^	0.5 (±0.1)	22 (±9.0)	2 (±0.4)	0.34 (±0.08)
Dd2attB+PfATP4^V178I^	2 (±0.3)	34 (±2.6)	17.4 (±4.0)	0.19 (±0.04)
Dd2::PfCDPK5^T392A^+PfATP4^wt^	0.8 (±0.07)	31 (±3.5)	2 (±0.01)	0.36 (±0.09)
Dd2::PfCDPK5^T392A^+PfATP4^V178I^	11 (±0.5)	176 (±8.0)	274 (±17)	0.62 (±0.07)

EC_50_, effective concentration for 50% growth inhibition; ND, not done.

EC_50_ values for three pyrazoleamides and a spiroindolone (NITD246) were derived from growth inhibition assays for Dd2 and a compound C2-1-resistant line, as well as five other transgenic lines derived from Dd2 parent. Growth inhibition curves are shown in Supplementary Fig. 5.

**Table 2:** Efficacy of antimalarials on *P. falciparum* grown in low [Na^+^] medium.

**Compound**	***P. falciparum*** **line**	**EC**_**50**_ **in normal [Na**^+^**] medium, nM (±s.e.m.)**	**EC**_**50**_ **in low [Na**^+^**] medium, nM (±s.e.m.)**
PA21A050	Dd2	0.5 (±0.01)	2.7 (±0.3)
PA21A085	Dd2	5.7 (±0.3)	19 (±0.9)
PA21A085	Dd2-21R	221 (±20)	>5,000
NITD246	Dd2	0.5 (±0.03)	1.8 (±0.1)
NITD246	Dd2-21R	0.7 (±0.02)	3.3 (±0.15)
Artemisinin	Dd2	11 (±0.4)	7 (±0.13)

EC_50_, effective concentration for 50% growth inhibition.

Growth inhibition assays were carried out for Dd2 and a compound 2-1 resistant line (Dd2-21R) using the indicated pyrazoleamide and a spiroindolone. The assays were carried out either in the normal growth medium or in a low [Na^+^] medium described in ref. 27.
