# Supplementary material for: Pyrazoleamide compounds are potent antimalarials that target Na+ homeostasis in intraerythrocytic Plasmodium falciparum
Source: Nat Commun. 2014 Nov 25;5:5521. doi: 10.1038/ncomms6521 (PMC4263321; doi:10.1038/ncomms6521)
Supplement: Supplementary Information — Supplementary Figures 1-13, Supplementary Tables 1-3 and Supplementary References [file ncomms6521-s1.pdf]

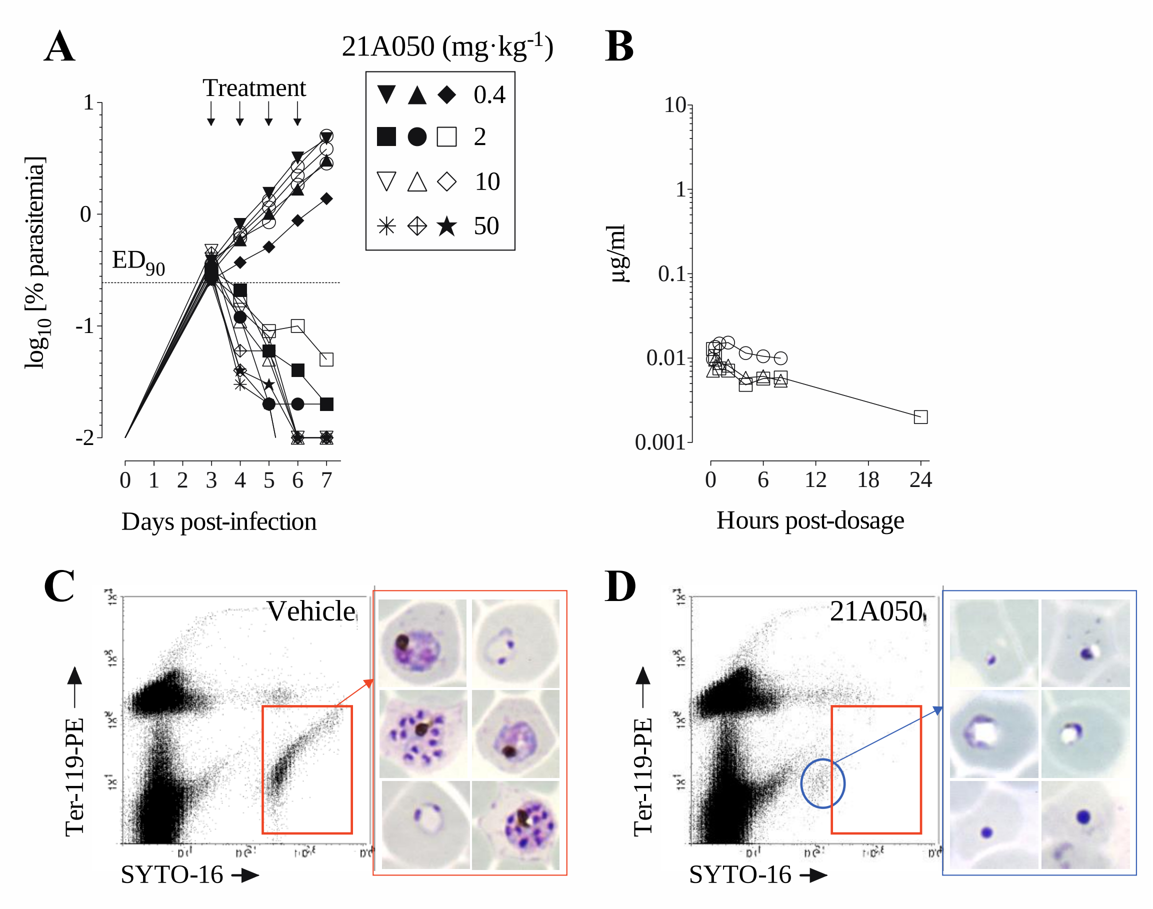

Supplementary Figure 1. ***In vivo* efficacy of PA21A050.** Four indicated doses of PA21A050 were administered orally to groups of 3 NOD/scid/IL2Rγ^{null} mice each engrafted with human erythrocytes and infected with *P. falciparum*. The compound was administered starting at day 3 post infection for 4 consecutive days. Parasitemia was assessed each day from day 3 post infection up to 7 days (A). Concentrations of PA21A050 were measured by LC/MS at 0.25, 0.5, 1, 2, 4, 7, 10, and 23 h after the first dose (B). Comparison of morphology of parasitized human RBC in vehicle treated (C) and PA21A050 treated (D) mice revealed parasites with only highly pyknotic staining in treated mice.

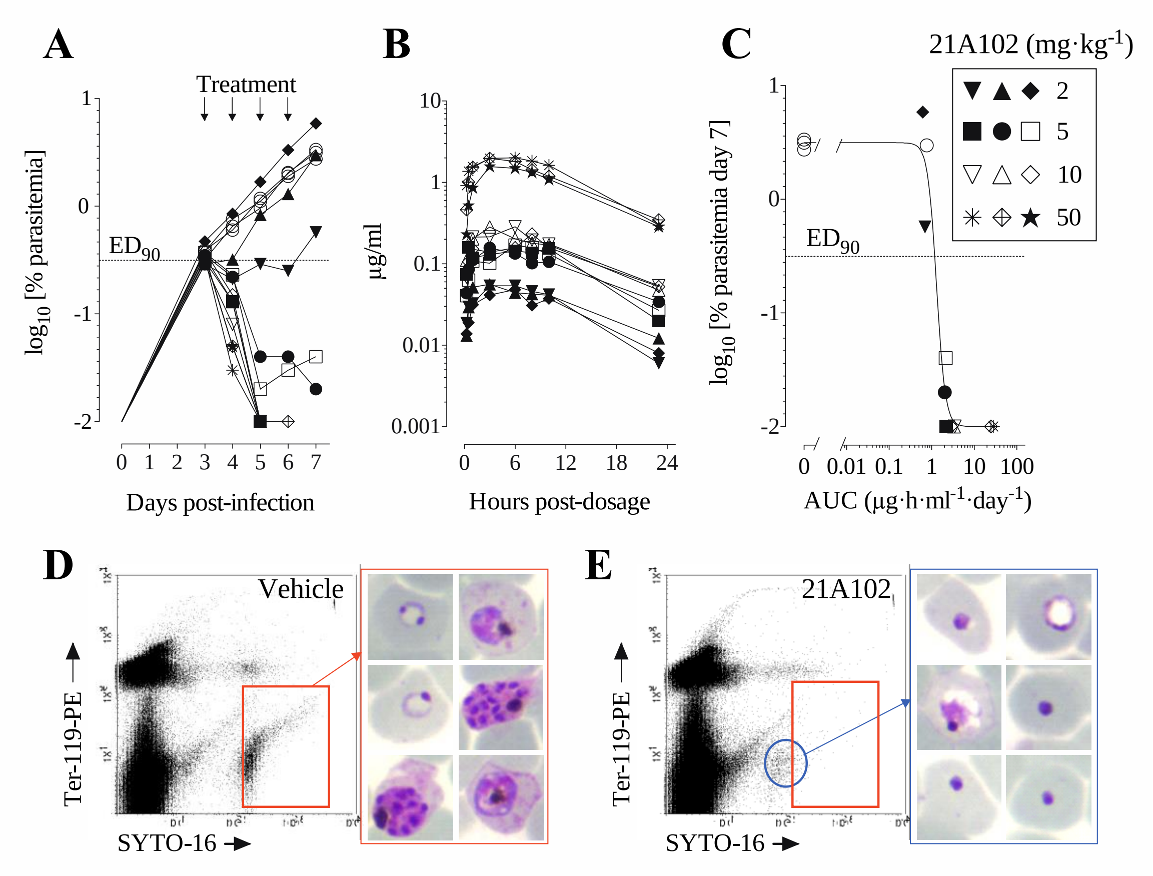

Supplementary Figure 2. *In vivo* efficacy of PA21A102. Four indicated doses of PA21A102 were administered orally to groups of 3 NOD/scid/IL2Rγ^{null} mice each engrafted with human erythrocytes and infected with *P. falciparum*. The compound was administered starting at day 3 post infection for 4 consecutive days. Parasitemia was assessed each day from day 3 post infection up to 7 days (A). Concentrations of PA21A102 were measured by LC/MS in each mouse at 0.25, 0.5, 1, 2, 4, 7, 10, and 23 h after the first dose (B). The estimated drug exposure necessary to inhibit *P. falciparum* parasitemia at day 7 post infection by 90% (AUC_{ED90}) was 0.24 µg.h.ml^{-1}.day^{-1} (C). Comparison of morphology of parasitized human RBC in vehicle treated (D) and PA21A102 treated (E) mice revealed parasites with only highly pyknotic staining in treated mice.

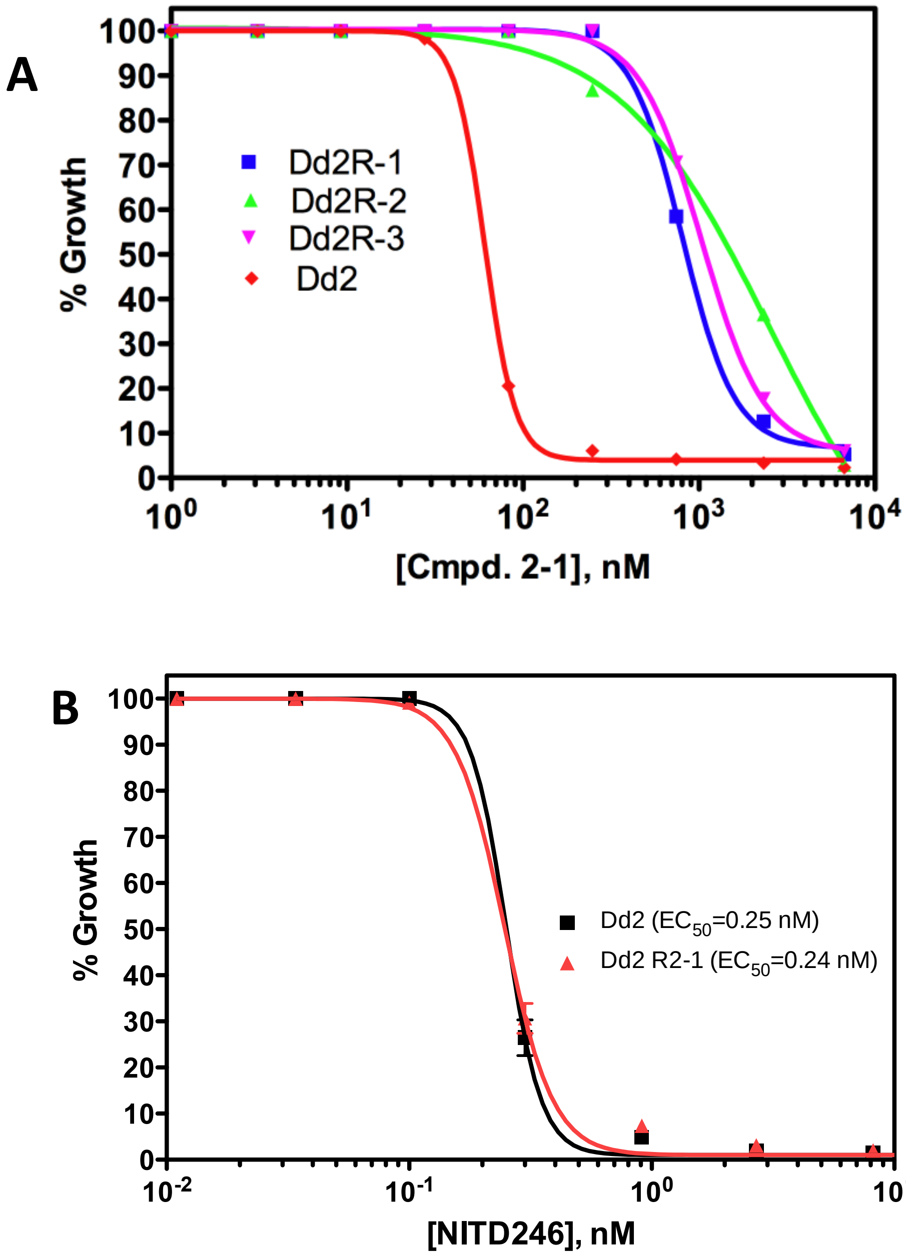

Supplementary Figure 3. Growth inhibition curves for compound C2-1 resistant parasites. (A). Growth inhibition curves for three independent lines derived from Dd2 parasites exposed to compound C2-1. Each of the resistant line had similar EC_{50} for compound C2-1 with some variation the curve slope. (B). Compound C2-1 resistant parasite line does not have cross-resistance to the spiroindolone NITD246. *P. falciparum* line Dd2 and its compound C2-1-resistant derivative, Dd2-R21, were used to test efficacy of the spiroindolone compound NITD246 in a growth inhibition assay.

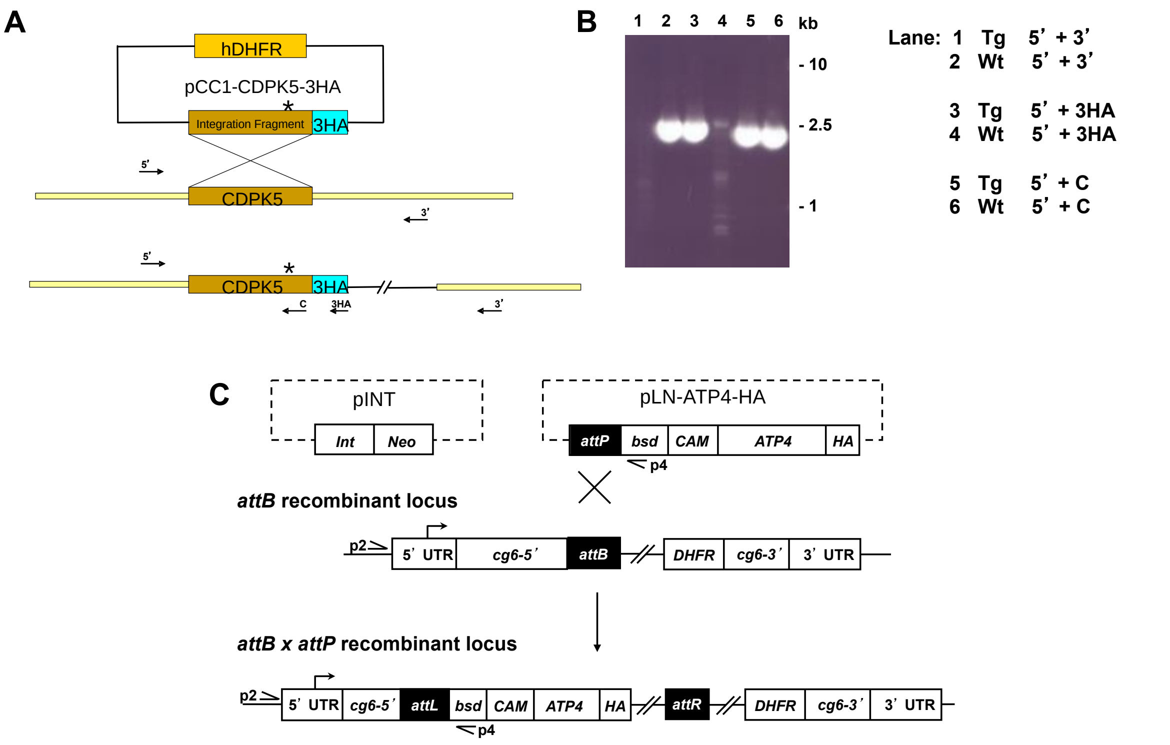

Supplementary Figure 4. Vectors and strategy for generating transgenic parasites (A) Schematic representation of the strategy used for replacing the PfCDPK5^{wt} allele with the PfCDPK5^{T392A} allele. (B) PCR confirmation of single cross over replacement by the PfCDPK5^{T392A} allele. Experimental details and the location of the specific primers used are presented in the SI Materials and methods. Tg, DNA from transgenic parasites; Wt, DNA from wild type parasite used for PCR amplifications. (C) Strategy to produce transgenic lines expressing wildtype or V178I mutant allele of PfATP4. Dd2attB line bearing attB sequence in the cg6 locus were co-transfected with pLN-ATP4-HA and pINT plasmids. The integrase encoded by pINT facilitates recombination between the attP sequence on pLN-ATP4-HA plasmid and the attB sequence, thereby generating the sequence shown at the bottom. Primer pairs P2 and P4 were used to confirm integration. We also used the same pLN-ATP4-HA plasmids (bearing the wildtype or mutant allele) to transfect Dd2::PfCDPK5^{T392A} parasites generated above. Here the plasmid was maintained with blasticidin drug pressure.

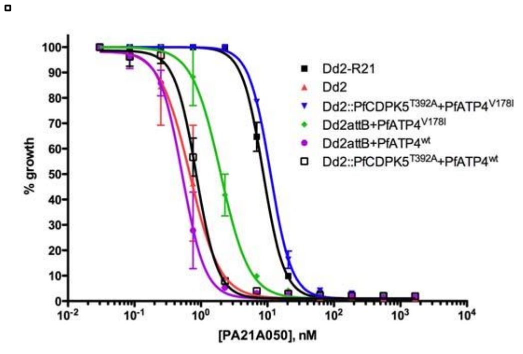

A

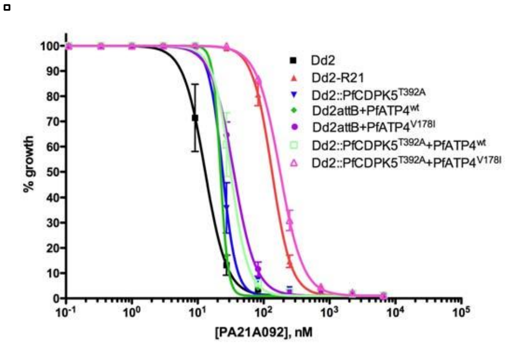

B

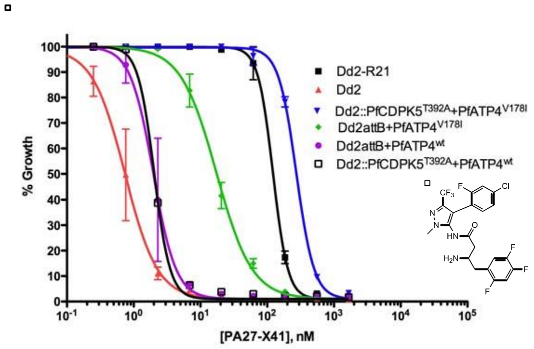

C

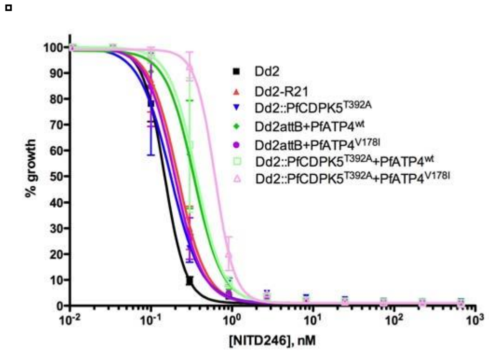

D

Supplementary Figure 5. Growth inhibitory curves for 4 compounds in Dd2 wildtype and various transgenic lines. Growth inhibition was assessed by ^{3}H-hypoxanthine incorporation in the indicated lines for pyrazoleamides PA21A050 (A), PA21A092 (B), PA27-X41 (C), and the spiroindolone NITD246 (D). The structure PA27-X41 is given in the inset of C.

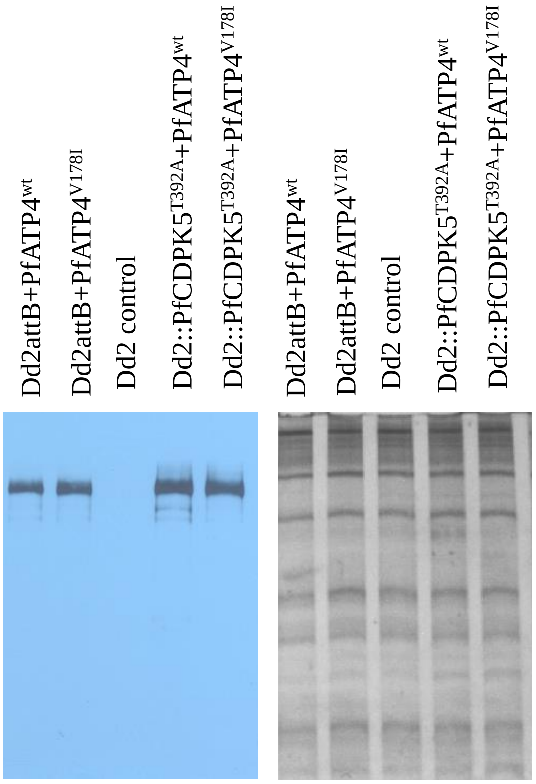

Supplementary Figure 6. Western blots for PfATP4 expression in transgenic parasite lines. Western blot of SDS-PAGE of the indicated parasite line was probed with an anti-HA tag antibody. Each of the transgenic line expresses PfATP4 with a 3xHA tag at its C-terminus. Left panel shows the Western blot and the right panel shows Coomasie Blue staining of the SDS-PAGE gel to indicate equal loading.

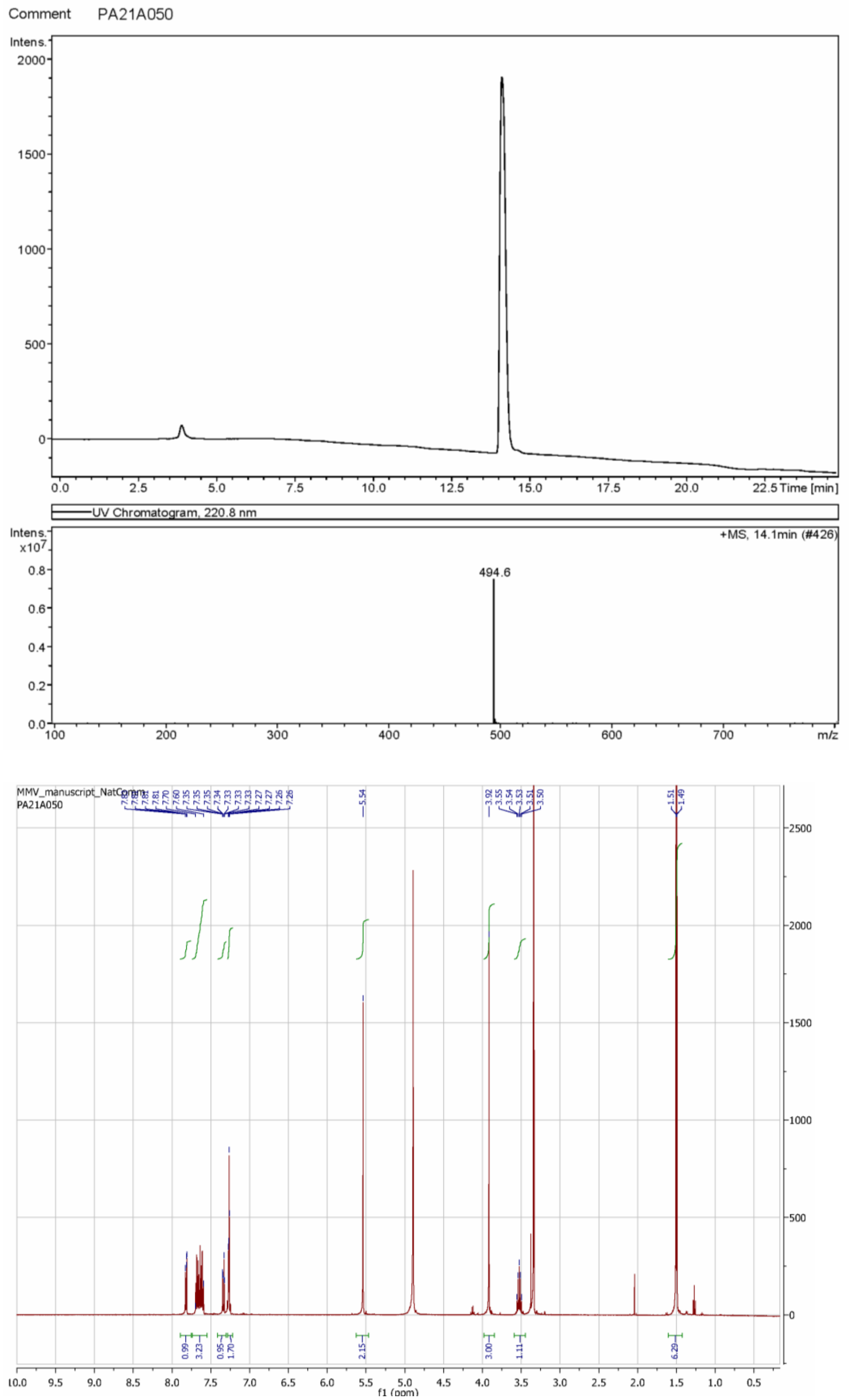

Supplementary Figure 7. LC-MS chromatogram (top) and ^{1}H NMR (bottom) of PA21A050.

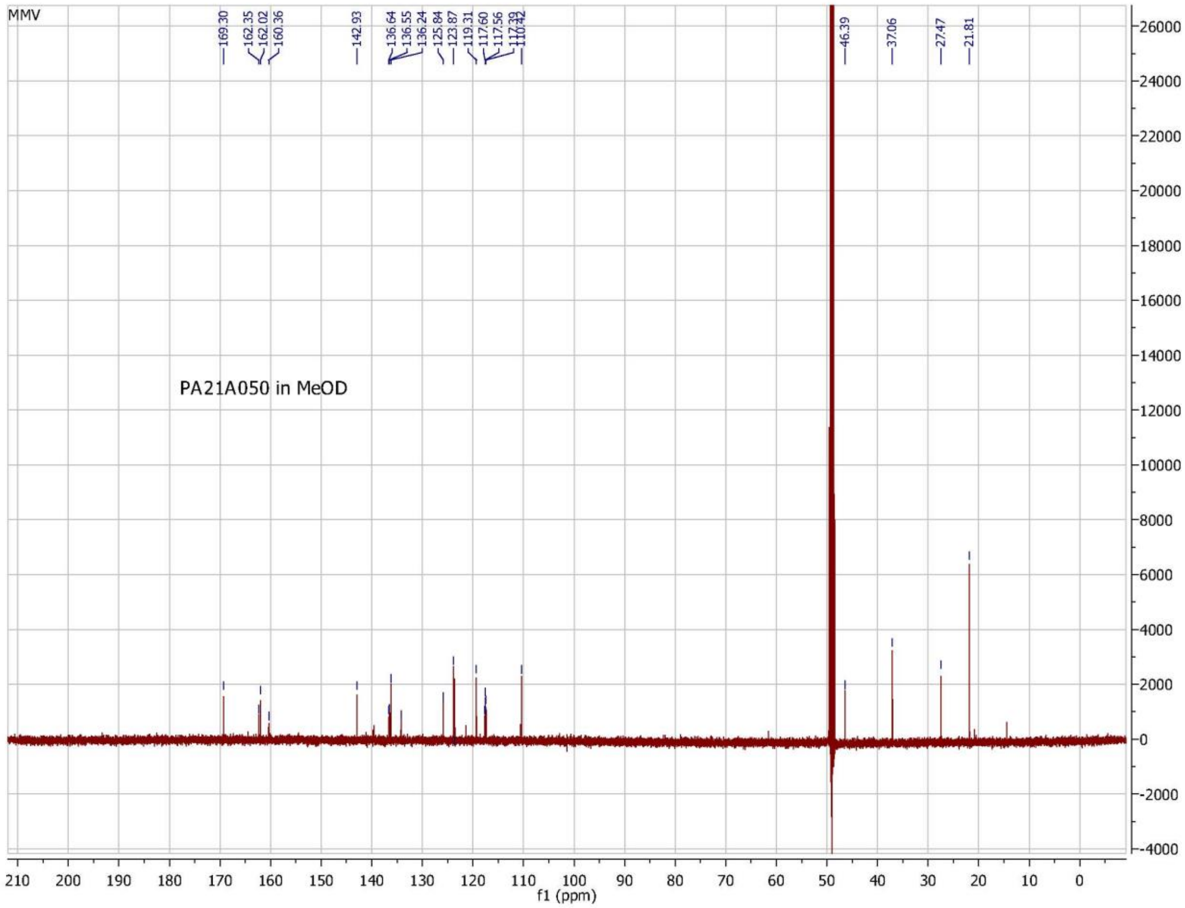

Supplementary Figure 8. ^{13}C-NMR spectra for PA21A050. The spectrum was obtained in MeOD at 126 MHz.

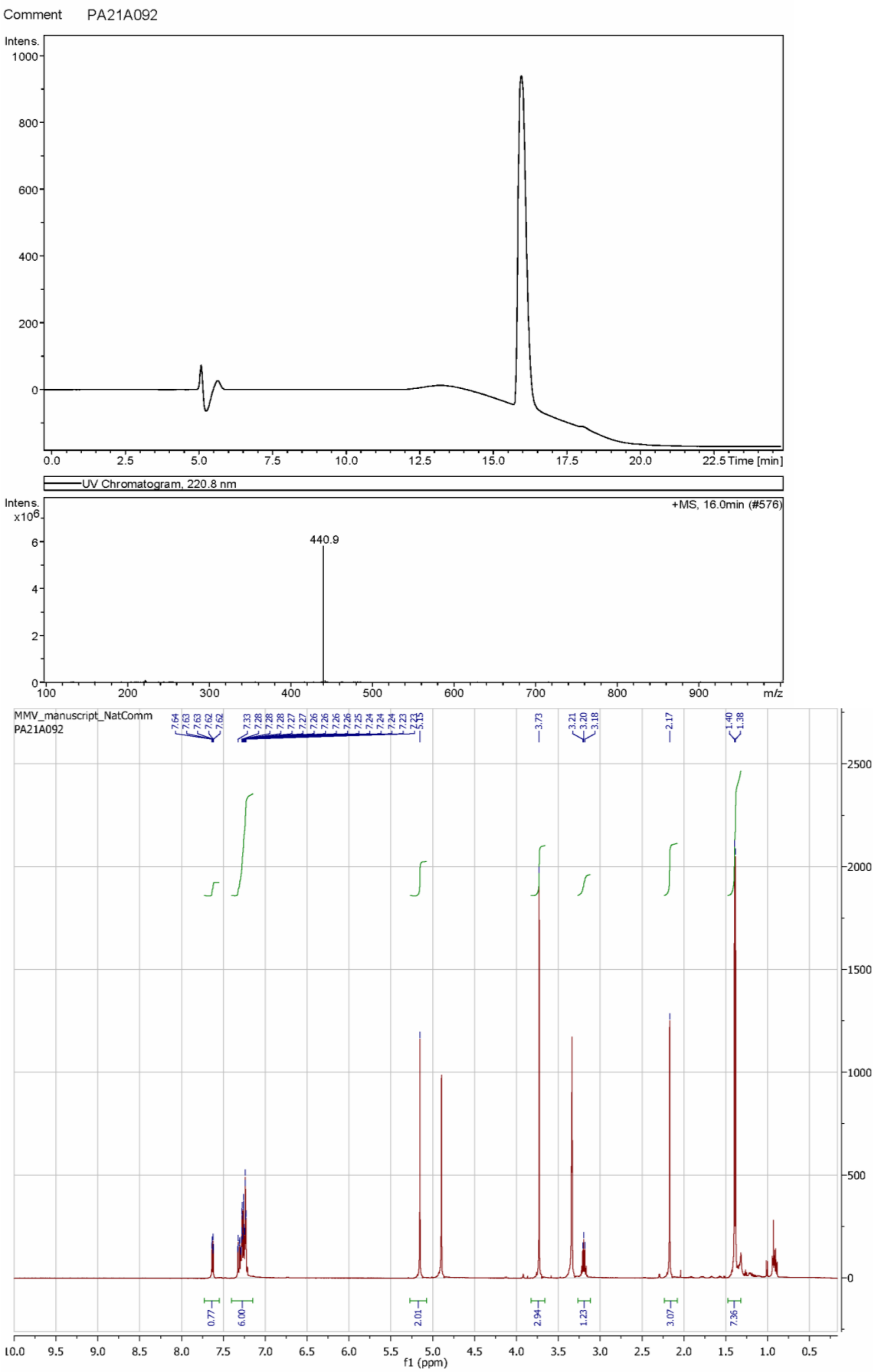

Supplementary Figure 9. LC-MS chromatogram (top) and ^{1}H NMR (bottom) of PA21A092.

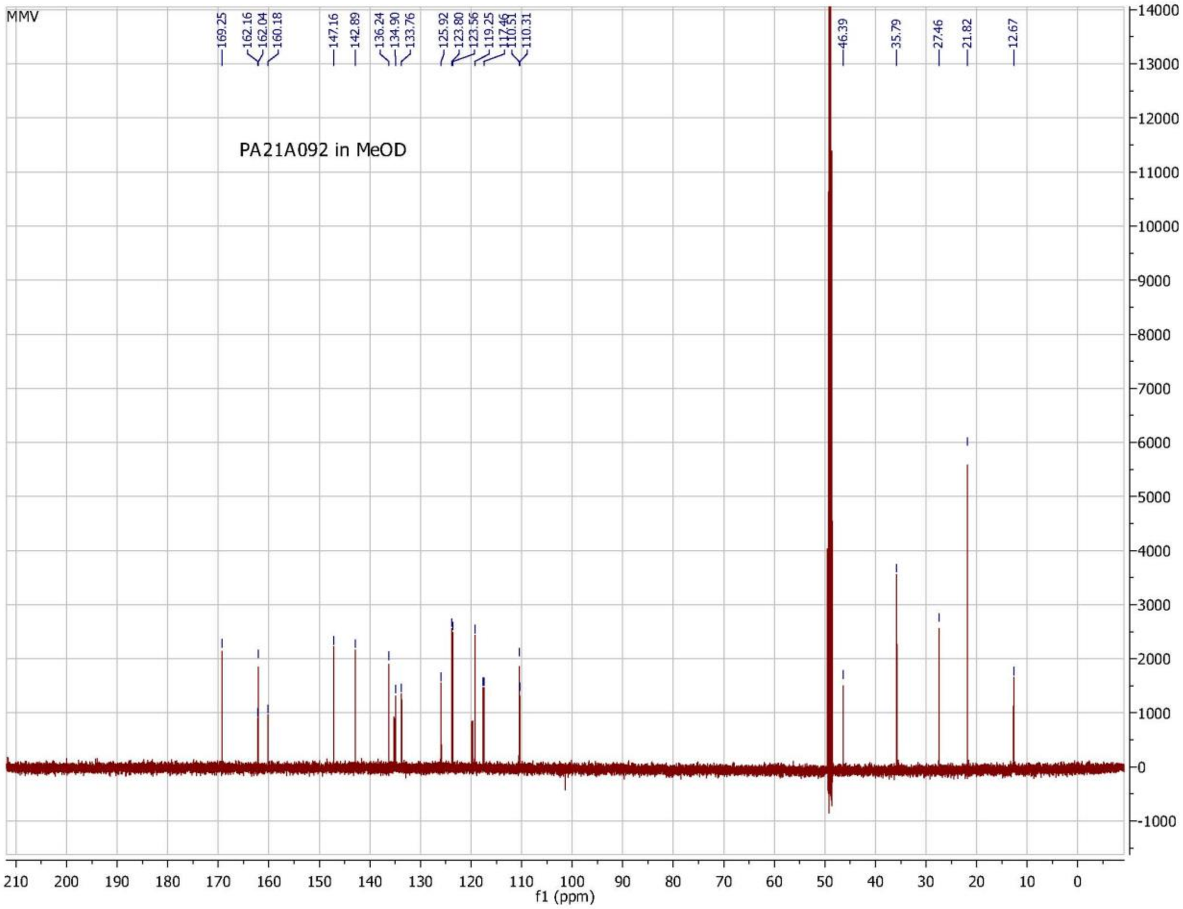

Supplementary Figure 10. ^{13}C-NMR spectra for PA21A092. The spectrum was obtained in MeOD at 126 MHz.

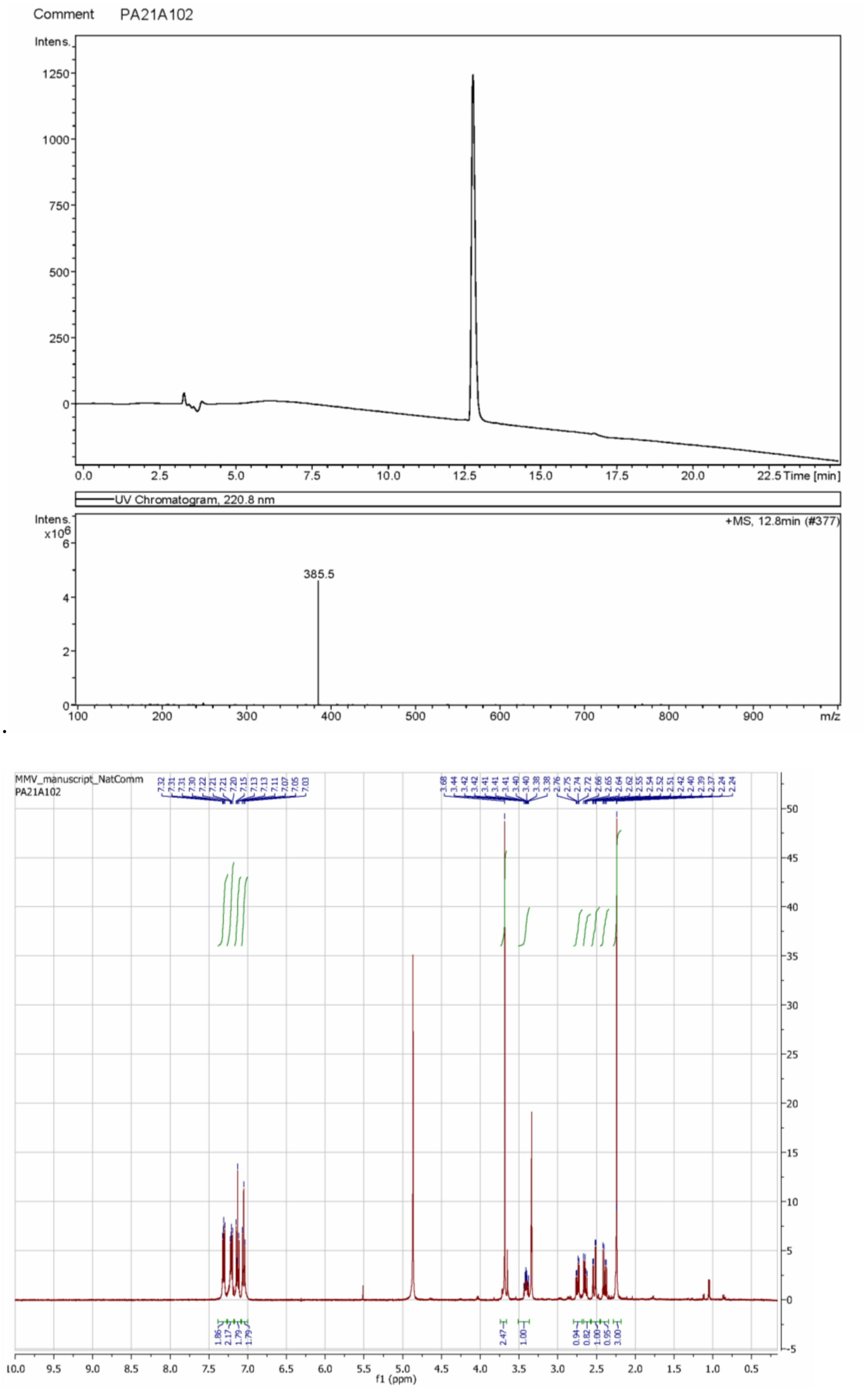

Supplementary Figure 11. LC-MS chromatogram (top) and ^{1}H NMR (bottom) of PA21A102.

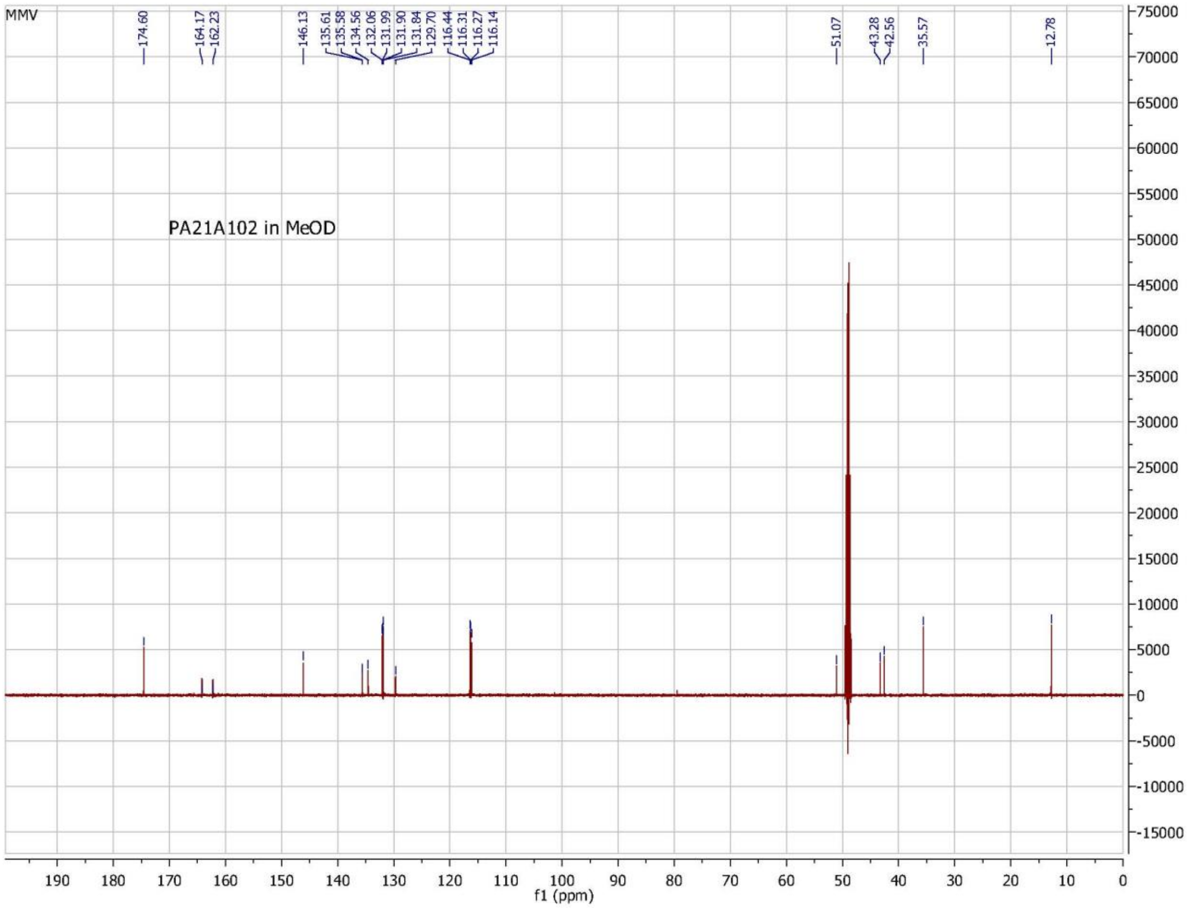

Supplementary Figure 12. ^{13}**C-NMR spectra for PA21A102.** The spectrum was obtained in MeOD at 126 MHz.

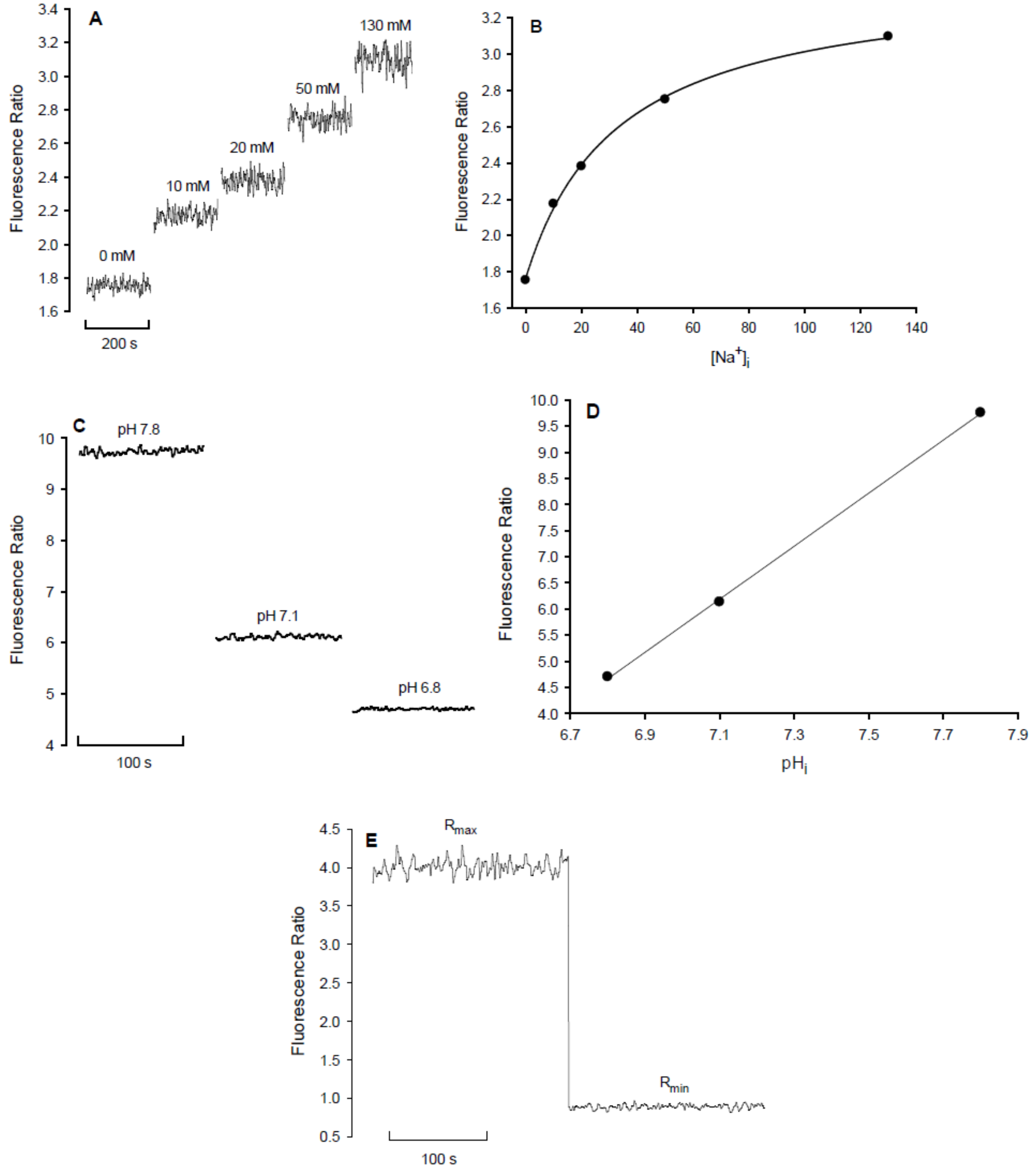

Supplementary Figure 13: Calibration of fluorescent ion-sensitive dyes

(A) Representative calibration traces for SBFI-loaded parasites in which $[Na^+]_i$ was equilibrated to $[Na^+]_o$, over a range of $[Na^+]_o$ values, using a combination of the ionophores gramicidin (2.5 µM), monensin (5 µM) and nigericin (5 µM). The traces, showing the fluorescence ratio (excitation at 340 nm/380 nm, with emission recorded at 515 nm) increasing with $[Na^+]_i$, are representative of those obtained in six independent experiments for this study and are similar to those obtained previously (see Supplementary Figure 1B in(1)). A separate calibration was performed for each experiment.

(B) Representative calibration curve, derived from the traces shown in **A**. For all such calibration curves the fluorescence ratio at each $[Na^+]_o$ was averaged over approximately 200 s and plotted as a function of $[Na^+]_i$ (equilibrated with $[Na^+]_o$) with the data fitted by a hyperbolic (three-parameter) rearrangement of the Grynkiewicz equation (1-3; see below). The Grynkiewicz equation is:

$[Na^+]_i = (K_d \times S_f/S_b) \times (R - R_{min}) /(R_{max} - R)$ *Equation 1*

where R is the emission fluorescence ratio (from excitation at 340 nm/380 nm) for the unknown $[Na^+]_i$, R_{min} is the emission ratio at 0 mM Na^+, R_{max} is the emission ratio at saturating Na^+ (achieved at 130 mM Na^+), K_d is the Na^+ dissociation constant of the dye, S_f is the fluorescence emission at 380 nm excitation for the free indicator (0 mM Na^+) and S_b is the fluorescence emission at 380 nm excitation for the Na^+-bound indicator (130 mM Na^+). Under physiological conditions K_d for SBFI is 17 - 19 mM (4, 5).

The calculation of the individual terms comprising K_dS_f/S_b is difficult, due to uncertainties in calculating a true value of S_b (2, 6). Therefore an alternative calibration approach, established by Diarra and colleagues (2001), was used, with the plot of fluorescence ratios as a function of $[Na^+]_i$ (derived from data as represented in Panel **A**) fitted by the following three-parameter hyperbolic equation rearranged from *Equation 1*:

$R = R_{min} \times (a \times [Na^+]_i)/(b + [Na^+]_i)$ *Equation 2*

where a and b are fitted constants ($a = R_{max} - R_{min}$ and $b = K_d \times S_f/S_b$) with all other terms as defined for *Equation 1*. From this equation, $[Na^+]_i$ for any R (unknown emission fluorescence ratio) can be calculated using the following rearrangement:

$[Na^+]_i = b \times (R - R_{min})/(R_{min} + a - R)$ *Equation 3*

where the terms are as defined for *Equations 1* and *2*.

In experiments in which the pH_i changed over the period in which $[Na^+]_i$ was measured, a correction factor was applied to distinguish between the changes in signal due to the pH-dependence of SBFI and the changes in the SBFI signal due to $[Na^+]_i$ changes The correction required was calculated as described previously (1, 2):

$[Na^+]_i$ (corrected) = ($[Na^+]_i$ (measured) × (7.3 – pH_{expt})) / σ *Equation 4*

where $[Na^+]_i$ (measured) is the $[Na^+]_i$ determined as per *Equation 3*, $[Na^+]_i$ (corrected) is the final value of $[Na^+]_i$ corrected for changes in pH_i, pH_{expt} is the pH_i (measured using the pH-sensitive dye BCECF-AM) and σ is an experimentally-determined constant. The "7.3" included in *Equation 4* is the resting pH_i of the mature *P. falciparum* trophozoite (7-11). σ was determined experimentally by performing the standard calibration at varying pH_o values (see Supplementary Figure 1C in (1)). An average σ of 1.73 ± 0.12 (n = 6) was determined and used throughout this study.

(C) Representative calibration trace for BCECF-loaded parasites in which pH_i was equilibrated to pH_o, over a range of pH_o values, using nigericin (30 μM). The traces, showing the fluorescence ratio (excitation at 495 nm/440 nm, with emission recorded at 520 nm) increasing with pH_i, are representative of those obtained in three independent experiments for this study and are similar to those described previously (7, 12, 13). A separate calibration was performed for each experiment.

(D) Representative calibration curve, derived from the traces shown in **C**. For all such calibration curves the fluorescence ratio at each pH_i was averaged over approximately 120 s and plotted as a function of pH_i (equilibrated with pH_o). The data were fitted by a straight line (typically $R^2 > 0.98$) which was then used to convert the measured fluorescence ratio to pH_i.

(E) Calibration trace for Fura-2-loaded parasites, representative of those obtained in three independent experiments for this study. At the end of each Fura-2 trace recorded in this study $CaCl_2$ and the Ca^{2+}-ionophore ionomycin (final concentrations 3 mM and 30 μM, respectively) were added to the cell suspension and incubated at 37 ˚C for 30 min. This allows an estimate of the maximum fluorescence ratio value (R_{max}, as in *Equation 1*). To determine R_{min}, 12.8 mM of the Ca^{2+}-chelator Ethylene glycol-bis(2-aminoethylether)-*N,N,N′,N′*-tetraacetic acid (EGTA) and 91 mM Tris(hydroxymethyl)aminomethane was added, and R_{min} (at both wavelengths) was recorded once fluorescence had stabilised. Fluorescence ratios were converted to $[Ca^{2+}]_i$ using the Grynkiewicz equation:

$[Ca^{2+}]_i = (K_d \times S_f/S_b) \times (R - R_{min}) /(R_{max} - R)$ *Equation 5*

where R is the fluorescence ratio (excitation at 340 nm/380 nm, with emission recorded at 515 nm) for the unknown $[Ca^{2+}]_i$, R_{min} is the fluorescence ratio at 0 mM Ca^{2+} (in the presence of

EGTA), R_{max} is the fluorescence ratio at saturating Ca^{2+} (achieved at 3 mM Ca^{2+}), K_d is the Ca^{2+} dissociation constant for the dye, S_f is the fluorescence emission at 380 nm excitation for the free indicator (0 mM Ca^{2+}) and S_b is the fluorescence emission at 380 nm excitation for the Ca^{2+}-bound indicator (3 mM Ca^{2+}). Under physiological conditions K_d for Fura-2 is approximately 140 nM (Molecular Probes Product information; revised Feb 2, 2011). This approach for calibration of Fura-2 loaded parasites (as used previously (14)) is different from that used for SBFI-/BCECF-loaded parasites (i.e. generation of a multi-point calibration curve as in **B**/**D**) and was taken due to the difficulties in preparing solutions of accurately-defined [Ca^{2+}].

Supplementary Table 1: Efficacy of PA21A092 against *P. falciparum* strains resistant to currently used antimalarial drugs.

Compound	*P. falciparum* Strain EC_{50} (nM)								
	**3D7A**	**Dd2**	**HB3**	**TM90C2A**	**TM90C2B**	**T9/94**	**V1/S**	**TC08**	**FCR3**
**PA21A092**	**18**	**22**	**32**	**14**	**13**	**13**	**38**	**28**	**19**
**Atovaquone**	1.0	3.0	2.0	3.0	>500	3.0	3.0	2.7	>500
**Pyrimethamine**	55	>20000	728	>20000	5354	53	>20000	>20000	90
**Artemisinin**	20	40	20	30	30	20	30	20	10
**Chloroquine**	20	440	30	520	760	430	800	50	430

Activity of 4 antimalarial drugs and PA21A092 were assessed in nine different strains of P. falciparum. Resistance profile of strains used is: **3D7** –sensitive; **HB3** – Pyrimethamine resistant; **Dd2** – Chloroquine, Mefloquine, Pyrimethamine and Sulfadoxine resistant; **Tm90C2A** – Chloroquine and Pyrimethamine resistant; **Tm90C2B** – Chloroquine, Atovaquone, Pyrimethamine and Cycloguanil resistant; **T9/94** – Chloroquine resistant; **V1/S** – Chloroquine and Pyrimethamine resistant; **TC08** – Pyrimethamine resistant; **FCR3** – Chloroquine, Atovaquone and Cycloguanil resistant.

Supplementary Table 2: Pharmacokinetic parameters of PA21A092 administered orally to NOD/scid/IL2R$_{\gamma}^{null}$ mice engrafted with human RBC and infected with *P. falciparum*.

Dose (mg/kg)	C_{max} (µg/ml)	T_{max} (h)	$AUC_{(0\text{-}t)}$ (µg.h/ml)*	$DNAUC_{(0\text{-}t)}$ (µg.h/ml per mg/kg)#
0.4	0.0055 (SD 0.00181)	2.8 (SD= 2.2)	0.0353 (SD= 0.0119)	0.0861 (SD= 0.029)
2.0	0.0268 (SD= 0.00678)	2.8 (SD= 2.0)	0.183 (SD= 0.0274)	0.0872 (SD= 0.013)
10	0.2 (SD= 0.0773)	0.33 (SD= 0.14)	0.896 (SD= 0.256)	0.0905 (SD= 0.0259)
50	0.759 (SD= 0.351)	1.5 (SD= 0.87)	5.56 (SD= 2.2)	0.113 (SD= 0.0449)

PA21A092 was orally administered to four groups of 3 mice each with the indicated dose. Blood samples of 25 µl were collected for determination of PA21A092 levels by LC/MS.

* t=10 h for the doses of 0.4, 2.0 and 10 mg/kg; t=23 h for the dose of 50 mg/kg.

# DNAUC, dose normalized value of $AUC_{0\text{-}t}$.

Supplementary Table 3: Compound C2-1-resistant *P. falciparum* parasites are cross-resistant to other active pyrazoleamide compounds.

Compound	*P. falciparum* EC_{50}, nM (±SEM)	
	Dd2	Dd2-R21
C416	150 (±20)	2882
C2-1	50 (±2.5)	697 (±30)
PA21A050	0.7 (±0.2)	16 (±0.5)
PA21A092	5.4 (±1.0)	135 (±8)
PA21A102	8 (±0.5)	264 (±35)
NITD246	0.15 (±0.01)	0.21 (±0.04)

Supplementary References

1 Spillman, N. J. *et al.* Na^+ Regulation in the Malaria Parasite *Plasmodium falciparum* Involves the Cation ATPase PfATP4 and Is a Target of the Spiroindolone Antimalarials. *Cell Host Microbe* **13**, 227-237 (2013).

2 Diarra, A., Sheldon, C. & Church, J. *In situ* calibration and $[H^+]$ sensitivity of the fluorescent Na^+ indicator SBFI. *Am J Physiol Cell Physiol* **280**, C1623-1633 (2001).

3 Grynkiewicz, G., Poenie, M. & Tsien, R. Y. A new generation of Ca^{2+} indicators with greatly improved fluorescence properties. *J Biol Chem* **260**, 3440-3450 (1985).

4 Minta, A. & Tsien, R. Y. Fluorescent indicators for cytosolic sodium. *J Biol Chem* **264**, 19449-19457 (1989).

5 Negulescu, P. A., Harootunian, A., Tsien, R. Y. & Machen, T. E. Fluorescence measurements of cytosolic free Na concentration, influx and efflux in gastric cells. *Cell Regul* **1**, 259-268 (1990).

6 Harootunian, A. T., Kao, J. P., Eckert, B. K. & Tsien, R. Y. Fluorescence ratio imaging of cytosolic free Na^+ in individual fibroblasts and lymphocytes. *J Biol Chem* **264**, 19458-19467 (1989).

7 Saliba, K. J. & Kirk, K. pH regulation in the intracellular malaria parasite, *Plasmodium falciparum:* H^+ extrusion via a V-type H^+-ATPase. *J Biol Chem* **274**, 33213-33219 (1999).

8 Hayashi, M. *et al.* Vacuolar H^+-ATPase localized in plasma membranes of malaria parasite cells, *Plasmodium falciparum*, is involved in regional acidification of parasitized erythrocytes. *J Biol Chem* **275**, 34353-34358, doi:10.1074/jbc.M003323200 [pii] (2000).

9 Kuhn, Y., Rohrbach, P. & Lanzer, M. Quantitative pH measurements in *Plasmodium falciparum*-infected erythrocytes using pHluorin. *Cell Microbiol* **9**, 1004-1013, doi:CMI847 [pii] 10.1111/j.1462-5822.2006.00847.x (2007).

10 Spillman, N. J., Allen, R. J. & Kirk, K. Acid extrusion from the intraerythrocytic malaria parasite is not via a Na^+/H^+ exchanger. *Mol Biochem Parasitol* **162**, 96-99, doi:S0166-6851(08)00170-9 [pii] 10.1016/j.molbiopara.2008.07.001 (2008).

11 Spillman, N. J., Allen, R. J. & Kirk, K. Na+ extrusion imposes an acid load on the intraerythrocytic malaria parasite. *Mol Biochem Parasitol* **189**, 1-4, doi:10.1016/j.molbiopara.2013.04.004 (2013).

12 Bray, P. G. *et al.* Cellular uptake of chloroquine is dependent on binding to ferriprotoporphyrin IX and is independent of NHE activity in *Plasmodium falciparum*. *J Cell Biol* **145**, 363-376 (1999).

13 Wunsch, S. *et al.* Differential stimulation of the Na^+/H^+ exchanger determines chloroquine uptake in *Plasmodium falciparum*. *J Cell Biol* **140**, 335-345 (1998).

14 Alleva, L. M. & Kirk, K. Calcium regulation in the intraerythrocytic malaria parasite Plasmodium falciparum. *Mol Biochem Parasitol* **117**, 121-128 (2001).
